# The role of APOBEC3C in modulating the tumor microenvironment and stemness properties of glioma: evidence from pancancer analysis

**DOI:** 10.3389/fimmu.2023.1242972

**Published:** 2023-09-21

**Authors:** Shoudu Zhang, Yugang Guo, Yuanzheng Hu, Xiaofang Gao, Fanghui Bai, Qian Ding, Kaiqi Hou, Zongqing Wang, Xing Sun, Hui Zhao, Zhongyu Qu, Qian Xu

**Affiliations:** ^1^ Henan Provincial Engineering Laboratory of Insects Bio-reactor, Nanyang Normal University, Nanyang, Henan, China; ^2^ The Department of Science and Technology, Zhengzhou Revogene Ltd, Zhengzhou, Henan, China; ^3^ Department of Oncology, Nanyang central Hospital, Nanyang, Henan, China

**Keywords:** A3 family, pan-cancer analysis, stemness, prognosis, tumor microenvironment

## Abstract

**Background:**

It is now understood that APOBEC3 family proteins (A3s) are essential in tumor progression, yet their involvement in tumor immunity and stemness across diverse cancer types remains poorly understood.

**Methods:**

In the present study, comprehensive genome-wide statistical and bioinformatic analyses were conducted to elucidate A3 family expression patterns, establishing clinically relevant correlations with prognosis, the tumor microenvironment(TME), immune infiltration, checkpoint blockade, and stemness across cancers. Different experimental techniques were applied, including RT–qPCR, immunohistochemistry, sphere formation assays, Transwell migration assays, and wound-healing assays, to investigate the impact of A3C on low-grade glioma (LGG) and glioblastoma multiforme (GBM), as well as its function in glioma stem cells(GSCs).

**Results:**

Dysregulated expression of A3s was observed in various human cancer tissues. The prognostic value of A3 expression differed across cancer types, with a link to particularly unfavorable outcomes in gliomas. A3s are associated with the the TME and stemness in multiple cancers. Additionally, we developed an independent prognostic model based on A3s expression, which may be an independent prognostic factor for OS in patients with glioma. Subsequent validation underscored a strong association between elevated A3C expression and adverse prognostic outcomes, higher tumor grades, and unfavorable histology in glioma. A potential connection between A3C and glioma progression was established. Notably, gene ontology (GO) and Kyoto Encyclopedia of Genes and Genomes (KEGG) analyses implicated A3C in immune system-related diseases, with heightened A3C levels contributing to an immunosuppressive tumor microenvironment (TME) in glioma. Furthermore, *in vitro* experiments substantiated the role of A3C in sustaining and renewing glioma stem cells, as A3C deletion led to diminished proliferation, invasion, and migration of glioma cells.

**Conclusion:**

The A3 family exhibits heterogeneous expression across various cancer types, with its expression profile serving as a predictive marker for overall survival in glioma patients. A3C emerges as a regulator of glioma progression, exerting its influence through modulation of the tumor microenvironment and regulation of stemness.

## 1 Introduction

The human APOBEC3 (apolipoprotein B mRNA editing catalytic polypeptide-like 3, A3s) gene family consists of seven cytidine deaminases (A3A, A3B, A3C, A3D, A3F, A3G, and A3H), which are located on chromosome 22 (1). Members of the A3 family reportedly play a role in the innate immune response to viral infections and can also cause damage to cellular DNA, potentially contributing to the initiation and progression of cancer (2, 3). Cellular overexpression of A3A can activate DNA damage responses and arrest the cell cycle (4). It is widely thought that the regulatory pathways of A3s might be influenced by viral infections (3). For instance, while initially thought to restrict HIV infection, A3A and A3G proteins can increase the viral mutational load, thereby reducing infectivity (5). High levels of A3B have been linked to human papillomavirus type 18 infection in cervical cancer (CESC) (6), as well as T-cell infiltration and improved outcomes in high-grade ovarian carcinoma (OV) (7). However, the precise functions and molecular mechanisms of A3s in cancer remain largely unclear, warranting further research.

Although cancer immunotherapy, especially checkpoint blockade immunotherapy, has shown promise in clinical outcomes, response rates remain modest (8, 9). The tumor microenvironment (TME) encompasses tumor cells, immune cells, stromal cells, cancer stem cells, and other cell types that collectively influence tumor progression, response to immunotherapy, and the emergence of drug resistance (10–12). A3s tend to accumulate in immune cells within the tumor microenvironment, affecting the development and response to immunotherapy in various cancers (13), including non-small cell lung cancer (NSCLC) (14, 15), head and neck cancer (HNC) (16), bladder urothelial carcinoma (BC), breast cancer (BRCA) (17, 18), and melanoma (19). Despite the mounting evidence associating A3s with the TME, the precise immune and stemness mechanisms of A3s across cancers remain poorly characterized.

Diffuse gliomas, encompassing low-grade glioma (LGG) and glioblastoma multiforme (GBM), represent the most aggressive brain tumors in adults, characterized by intricate pathogenic mechanisms and a propensity for relapse (20). Patients diagnosed with LGG, typically exhibit more favorable prognoses in contrast to those diagnosed with GBM. Nevertheless, it is important to note that a significant number of LGG cases eventually progress to GBM (21). To date, after receiving optimal treatment, the 5-year survival rate of GBM is still less than 5% and the median OS of LGG is less than 2 years (22), emphasizing the need for novel therapeutic approaches. In recent years, immunotherapy has exhibited promising outcomes in cancer treatment, shedding new light on the clinical management of glioma (23).

Herein, we comprehensively analyzed A3s expression across normal tissues, cell lines, and pancancer samples, and explored the associations between A3s expression and tumor prognosis, TME characteristics, and stemness characteristics. We investigated the role of A3C in modulating the TME and influencing stemness properties, particularly within the context of glioma. The findings from our investigation have the potential to yield valuable insights for fundamental scientific inquiries and contribute to the advancement of therapeutic strategies targeting A3C for anticancer therapeutic purposes.

## 2 Materials and methods

### 2.1 Data collection and bioinformatics analysis

We obtained various data types, including gene expression profiles, tumor mutational burden (TMB) data, microsatellite instability (MSI) data, mismatch repair (MMR) data, DNA methylation data, RNA stemness scores (RNAss), DNA stemness scores (DNAss), and clinical attributes for 33 cancer types from the UCSC Cancer Genome Browser (https://tcga.xenahubs.net). Expression data for A3s were obtained from The Genotype-Tissue Expression (GTEx)(https://www.gtexportal.org), The Cancer Genome Atlas (TCGA), and Cancer Cell Line Encyclopedia (CCLE) (http://portals.broadinstitute.org/ccle). Independent validation glioma datasets (GSE16011), and single-cell glioma datasets (GSE185231 and GSE136974), were retrieved from GEO (https://www.ncbi.nlm.nih.gov/geo/). Differential expression and prognostic correlations were analyzed using the SangerBox online platform (http://sangerbox.com/). Forest plots were constructed based on hazard ratio (HR) distributions and corresponding 95% confidence intervals. We selected the “TCGA Pan-Cancer Atlas Studies” datasets for examining A3 genetic alterations through the cBioPortal website (http://www.cbioportal.org/). TMB and MSI score were calculated from TCGA somatic mutation data, with Spearman’s correlation assessing the potential relationship between A3s expression and TMB/MSI. Tumor microenvironment analysis involved calculating the stromal score, immune score, and ESTIMATE score using the ESTIMATE package (24). Immune infiltration levels were determined using Tumor Immune Estimation Resource (TIMER) (25) and EPIC algorithms (26). Spearman correlation analysis was performed using the R package cor to determine whether the expression of A3s genes was associated with RNAss or DNAss (27). A prognostic model was constructed using glioma datasets from TCGA and the Chinese Glioma Genome Atlas (CGGA; https://www.cgga.org.cn/) as training and validation datasets, respectively. The glioma prognostic model was constructed by multivariate Cox regression analysis, and survival analysis was performed with the R statistical package. Analyses of coexpression, protein–protein interactions, and functional genetic interactions were performed with GeneMANIA (http://genemania.org/). The identification of hub genes in the protein interaction network was conducted using the cytoHubba plugin of Cytoscape. Subsequently, survival analysis and clinicopathological correlation analysis of A3C and A3G were performed in glioma datasets from TCGA and CGGA datasets available on GlioVis data portal (http://gliovis.bioinfo.cnio.es/). The correlation between A3C protein expression and glioma clinicopathology was investigated by conducting an analysis using the Clinical Proteomic Tumor Analysis Consortium (CPTAC) database available on the University of Alabama at Birmingham Cancer Data Analysis Portal (UALCAN) website (https://ualcan.path.uab.edu/). For A3C-related gene enrichment analysis, we searched the top 300 A3C-correlated targeting genes by using Gene Expression Profiling and Interactive Analyses (GEPIA2; http://gepia2.cancer-pku.cn) database base on TCGA-GBM, TCGA-LGG, and GTEx normal brain tissue datasets. Then Kyoto Encyclopedia of Genes and Genomes (KEGG) pathway and Gene Ontology (GO) term enrichment analyses of A3C-related genes were performed using the SangerBox portal. Correlations between A3s expression and stemness features were determined using GEPIA2.

### 2.2 Patients and variables

We conducted a prospective study from August 2021 to August 2022. The study incorporated blood samples obtained from 12 patients diagnosed with GBM, blood samples obtained from 12 healthy controls, and 6 paraffin-embedded glioma tissue samples (3 cases of LGG and 3 cases of GBM). All experimental procedures adhered to the guidelines and regulations set forth by the Ethics Committee of Nanyang Central Hospital. Prior to their inclusion, all participants or their legal representatives provided informed consent to participate in this study.

### 2.3 Cell culture

The Chinese Academy of Medical Sciences (Shanghai, China) provided two human glioma cell lines (U251 and Hs683). Cells were cultured in Dulbecco’s modified Eagle’s medium (DMEM) supplemented with 10% fetal bovine serum (FBS) and 1% penicillin−streptomycin solution, under standard conditions. For the preparation of GSC-U251 and GSC-Hs683 cells, the glioma cells were grown in serum-free DMEM/F12 media containing epidermal growth factor (EGF, 20 ng/mL), basic fibroblast growth factor (bFGF, 20 ng/mL), B27 supplement, and 1% penicillin−streptomycin solution. The cells were cultured at 37°C, in 5% CO_2_, and 95% humidity.

### 2.4 Lentiviral transduction and construction of stable cell lines

A lentivirus-based packaging system was developed using the plvx-shRNA2-ZSGreen-T2A-puro lentiviral vector (Oligobio, Beijing, China). The shRNA-APOBEC3C and scramble-shRNA controls (sh-NT) sequences were synthesized by Oligobio (Beijing, China). At a confluence of 30%-50%, cells were transduced with lentiviral particles at a multiplicity of infection of 50 and incubated for 48 h. The medium was replaced by a fresh complete medium, and 2 µg/mL of puromycin was added to eliminate uninfected cells. The infection efficiency was assessed by fluorescence microscopy, and stable integration was validated by RT–qPCR.

### 2.5 Total RNA extraction and quantitative real-time PCR

RNA was extracted from peripheral blood samples and stored in blood RNA storage tubes (BioTeke Corporation, Beijing, China). Total RNA was converted to cDNA using an M5 Superplus RT–qPCR kit with gDNA (Mei5 Biotechnology Co. Ltd., Beijing, China). The CFX96TM System (Bio-Rad, CA, USA) was used for RT–qPCR analysis. The primer sequences were A3C-F: CTCTTCCAGACTCTTCCTG and A3C-R: GGATGAGTTTAGTAAGATTTGGTT. GAPDH was used as an internal reference gene.

### 2.6 Immunohistochemistry

First, paraffin-embedded sections (2 µm-thick) of glioma tissue of various grades were prepared using conventional dehydration and paraffin embedding procedures. In brief, paraffin sections were baked in citrate buffer (1 h, 65°C), washed with water, and subjected to high-pressure antigen retrieval. Endogenous peroxidase activity was blocked by incubation for 10 min with 3% H_2_O_2_. After blocking, the sections were incubated with an anti-A3C antibody (1:100; 10591-1-AP, Proteintech, USA) at 37°C for 1 h. The sections were washed 5 times with PBS and were then incubated with a secondary antibody (polyclonal rabbit IgG, Abcam). After staining with diaminobenzidine (DAB), the sections were washed with PBS to remove excess stain, counterstained with hematoxylin, differentiated with hydrochloric acid alcohol, and blued with tap water. Finally, the sections were dehydrated through an alcohol gradient, mounted in neutral resin in xylene, and observed under an M2000 LED microscope (Leica, Germany). Images were processed using Motic DSAssistant software and staining was quantified by measurement of the optical density using ImageJ software (v1.46, Bethesda, MD, USA).

### 2.7 Sphere formation assay

The human glioma cell lines, U251 and Hs683, were cultured to 80% confluence, followed by trypsin digestion to obtain a suspension of individual cells. The cells were washed twice with PBS and resuspended in serum-free DMEM/F12 medium supplemented with EGF (20 ng/mL), b-FGF (20 ng/mL), and B27 supplement. Subsequently, the cells were seeded into 6-well ultra-low-attachment plates (Corning, USA). After an incubation period of 8-10 days, the formed spheres were quantified using a light microscope (100×), following previously established methods. The cell culture conditions included maintenance at 37°C and 5% CO_2_, as described in a previous study (28).

### 2.8 Cell viability assays

Cells were seeded into 96-well plates, and their viability was assessed at 0, 24, and 48 h post-transfection. To conduct the CCK assay, 10 μL of CCK-8 solution (Dojindo Laboratories, Japan) was added to the wells, and the plates were incubated for 2 h. Subsequently, the absorbance was measured at a wavelength of 450 nm. The relative cell viability(%) was determined by comparing the viability in the treated group to that in the untreated control group (29).

### 2.9 Invasion assay and wound healing assay

Corning Transwell chambers coated with Matrigel (diluted 1:8; BD Biosciences, Bedford, MA, USA) were used for the cell invasion assay. Cells were added to the upper compartment as suspensions of 5 × 10^4^ cells per well (120 μL) in serum-free medium, while 600 μL of medium containing 10% FBS was added to the lower compartment. After methanol fixation and crystal violet staining, an inverted microscope (EVOS, AMG, USA) was used to observe cells that migrated to the lower surface of the membrane. Wound healing assay was performed in 6-well plates. At 80% confluence, the monolayer was scraped off using a sterile pipette tip. Inverted fluorescence microscope images were obtained and analyzed using ImageJ.

### 2.10 Statistical analysis

Spearman or partial Spearman methods were used to examine correlations between genetic data and RNAss, DNAss, immune score, stromal score, ESTIMATE score, TMB, immune infiltration, immune checkpoint genes expression, and tumor stemness markers expression. All experiments were performed in triplicate. Two-tailed t tests and one-way analysis of variance (ANOVA) were used to assess the significance of differences between groups. Statistical significance was defined as P< 0.05. In addition to online analysis, all data analyses were conducted in R (v. 3.6.1, http://www.r-project.org/) and GraphPad Prism (v.8.0, GraphPad Software, San Diego, CA, USA).

## 3 Results

### 3.1 Abnormal expression of A3s genes in various healthy and cancerous human tissues

**Figure 1:**
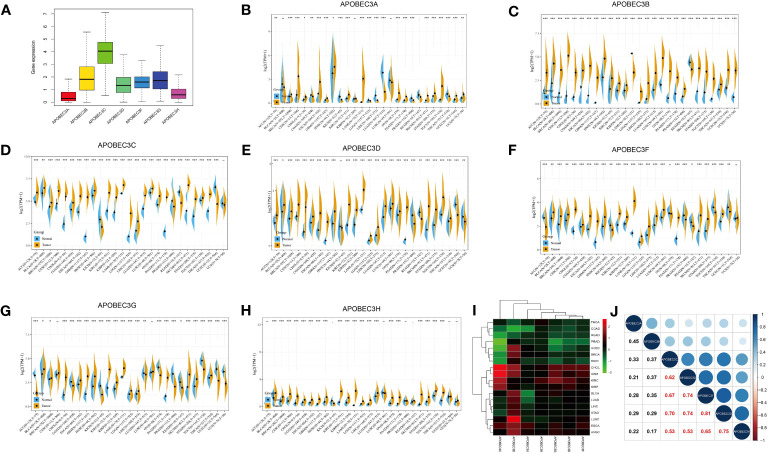
Differential expression of A3s between cancer and healthy tissues. **(A)** Boxplot showing the relative transcript levels of A3s in 33 cancer types. **(B–H)** A3s expression in normal tissues (TCGA and GTEx) and cancer tissues (TCGA). **(I)** Heatmap showing A3s expression differences between primary tumors and adjacent normal tissues. **(J)** Correlation plots and coefficients for A3s. *P< 0.05, **P< 0.01, ***P< 0.001.

Among the 33 cancer types in TCGA pancancer database, the A3 family genes exhibited varying expression levels, with A3C showing the highest relative expression (**Figure 1A**). Using the GTEx database, we assessed A3s expression in normal tissues and found that all seven A3 genes exhibited higher expression in blood (n=444) and lower expression in brain tissue (n=1152) than in other tissues (**Figure S1**). Integrated analysis of the GTEx and TCGA databases showed that A3s are differentially expressed in cancer tissues and the corresponding control tissues in most cancer types (**Figures 1B–H**, P< 0.05). Notably, A3s exhibited elevated expression levels in several cancer types, including GBM, cholangiocarcinoma (CHOL), colon adenocarcinoma (COAD), head and neck squamous cell carcinoma (HNSC), liver hepatocellular carcinoma (LIHC), lung adenocarcinoma (LUAD), lung squamous cell carcinoma (LUSC), LGG, stomach adenocarcinoma (STAD), kidney renal clear cell carcinoma (KIRC), and pancreatic adenocarcinoma (PAAD) tissues than in the corresponding normal tissues. We also detected high A3s expression in most cancer cell lines (**Figure S2**) and found significant heterogeneity in A3s expression between tumor types (**Figures 1B–H**, P< 0.05). High A3C expression was found in CHOL, GBM, KIRC, and kidney renal papillary cell carcinoma (KIRP), while exhibiting low expression in COAD, rectum adenocarcinoma (READ), prostate adenocarcinoma (PRAD), uterine corpus endometrial carcinoma (UCEC), BRCA, and kidney chromophobe (KICH). Moreover, A3B displayed high expression in UCEC, BRCA, CHOL, GBM, BLCA, LUAD, LIHC, LUSC, and esophageal carcinoma (ESCA), with low expression in thyroid carcinoma (THCA), COAD, and READ. These findings collectively indicate the presence of distinct patterns of A3s expression in different cancer types (**Figure 1I**). Moreover, Pearson’s correlation analysis revealed significant correlations between expression levels of A3 family members across cancers (**Figure 1J**, P<0.01). Notably, specific pairs of A3 family members exhibited significant correlations such as A3C×A3D (r=0.62), A3C×A3F (r=0.67), A3C×A3G (r=0.70), A3D×A3F (r=0.74), A3D×A3G (r=0.74), and A3F×A3G (r=0.81).

### 3.2 Prognostic potential of A3s expression across human cancers

**Figure 2:**
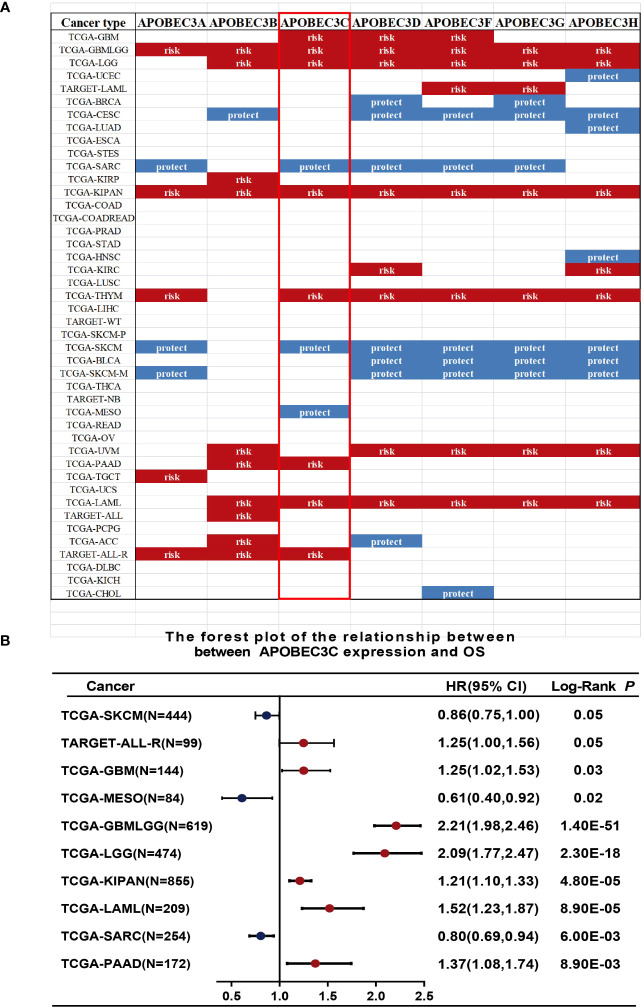
Relationship between A3s expression and OS. **(A)** Expression of A3s identified as risk or protective factors in the TCGA and GTEx datasets. **(B)** Forest plot relating A3C expression to OS across 9 tumors.

We further investigated the prognostic implications of dysregulated A3s expression in pancancer by merging TCGA and GTEx databases. Our analysis revealed that all seven A3 genes were identified as risk factors in the GBMLGG and the pankidney cohort (KIPAN) (**Figure 2A**). Importantly, A3C exhibited a hazard ratio greater than 1 and was identified as an adverse prognostic gene in glioma, KIPAN, thymoma (THYM), PAAD, and LAML, while serving as a protective prognostic gene in three tumor types (sarcoma (SARC), SKCM, and mesothelioma (MESO)) (**Figure 2B**). In addition, forest plots illustrated the relationship between A3s expression and OS across cancers, with high A3D, A3F, A3G, and A3H expression associated with increased risk in specific tumor types, while acting as protective factors in others (**Figure S3**). Thus, the prognostic significance of A3s expression varies depending on cancer type, with a link to particularly unfavorable outcomes in glioma.

### 3.3 Genetic alteration analysis and correlation analysis of A3s expression level with TMB score, MSI score, MMR gene expression, and DNA methylation level

**Figure 3:**
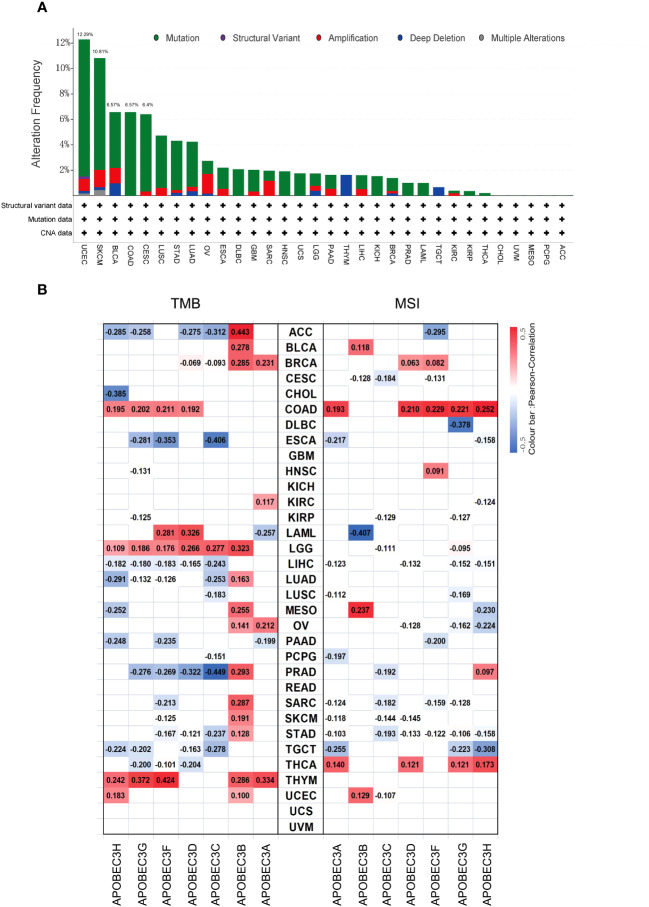
Genetic alteration frequency of A3s and correlations of A3s with TMB and tumor MSI. **(A)** Genetic alteration frequency of A3s in different types of tumors. **(B)** Correlations of A3 expression with TMB and MSI in multiple cancers.

Subsequently, considering that distinct mutations or genetic variations influence the initiation and progression of tumors, the genomic profiling data obtained from cBioPortal were analyzed with respect to the 32 cancer types represented in the TCGA database (consisting of 10,967 samples) to assess the frequency of genetic alterations in A3 genes. As illustrated in **Figure 3A**, the frequency of genomic alterations in A3s genes was notable, with rates of 12.29% (529 cases) in UCEC, 6.57% (411 cases) in SKCM, 6.57% (411 cases) in BLCA, 6.57% (594 cases) in COAD, and 6.4% (297 cases) in CESC, emerging as the top five alteration frequencies for A3s genes (**Figure 3A**). Furthermore, among the A3s genes, the highest occurrence of genetic alterations, specifically in A3D and A3F (>5% in 529 cases), was observed in UCEC patients (**Figure S4**).

Given prior research suggesting that elevated TMB and MSI levels can enhance antitumor responses of lymphocytes and promote immune recognition of tumors (30–32). we aimed to explore potential correlations between A3s expression levels and TMB and MSI. The relationship between A3s expression and TMB/MSI is depicted in **Figure 3B**. Importantly, a positive correlation was found between A3B expression and TMB in 15 out of the 33 cancer types, including adrenocortical carcinoma (ACC), BLCA, BRCA, LGG, LUAD, MESO, ovarian serous cystadenocarcinoma (OV), PRAD, SARC, SKCM, STAD, and THYM (P < 0.05). Conversely, A3C expression exhibited a significant negative correlation with TMB in ESCA and PRAD, with the highest correlation coefficients. In terms of MSI, A3G displayed a negative association with MSI in DLBC, having the lowest correlation coefficient, while showing a positive relationship with MSI in COAD. Interestingly, A3B expression exhibited positive correlations with both TMB and MSI in MESO. Additionally, as shown in **Figure S5**, a significant correlation was observed between A3B/A3C/A3F expression and MMR genes in eleven distinct cancer types (ACC, ESCA, HNSC, KICH, KIRC, KIRP, LGG, LIHC, LUAD, PAAD, and STAD). Given that aberrant DNA methylation is recognized for inducing alterations in gene expression that contribute to cancer progression, we found that the expression of A3s were positively correlated with that of DNA methyltransferases (DNMT1/2/3A/3B) across various cancer types, including COAD, HNSC, KIRP, KICH, KIRC, LGG, LIHC, CESC, LUAD, PAAD, STAD, LUSC, THCA, and testicular germ cell tumors (TGCT), as shown in **Figure S6** (P < 0.05).

### 3.4 A3s are associated with the immune microenvironment and stemness across human cancers

**Figure 4:**
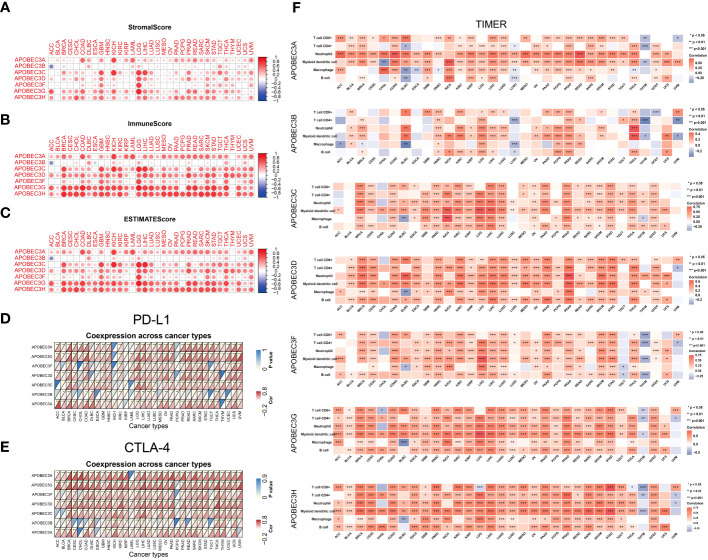
Relationships between A3s expression and the TME. Correlation of A3 expression levels with the **(A)** stromal score, **(B)** immune score and **(C)** ESTIMATE score in patients across cancers. **(D, E)** Heatmaps of associations between A3 expression and PD-L1 and CTLA-4 expression across cancers. **(F)** Heatmap of the Spearman correlation analysis between the immune infiltration score determined with TIMER and A3 expression in multiple tumor tissues. *P<0.05, **P< 0.01, ***P< 0.001.

Next, we explored the correlation between A3s genes expression and immune, stromal, and ESTIMATE scores, whereby higher scores were associated with more substantial immune or stromal infiltration within the TME (24). Consequently, a positive association emerged between the expression of A3s genes, particularly A3A, A3C, A3D, A3G, and A3H, and the TME scores (including immune, stromal, and ESTIMATE scores) across a range of tumor types, with a particularly notable effect observed in LGG (as illustrated in **Figures 4A–C**, P < 0.05). The significance of stromal and immune cells in cancer progression, metastasis, and resistance to treatments has been well-established, suggesting that A3s genes could influence tumor behavior through interactions with the tumor microenvironment. Moreover, we identified substantial positive correlations between the expression of A3s genes and immune checkpoint genes (CTLA-4 and PD-L1) in the majority of tumor types, including BRCA, CESC, GBM, LGG, HNSC, KIRC, THCA, and USC (as depicted in **Figures 4D, E**; P < 0.05). Furthermore, we assessed immune infiltration scores using the TIMER and Estimating the Proportions of Immune and Cancer cells (EPIC) algorithms across various cancer types. The results displayed positive associations between A3s expression and immune responses, as well as tumor infiltration by diverse immune cell types such as macrophages, CD4^+^ T cells, CD8^+^ T cells, neutrophils, myeloid dendritic cells, and B cells (P<0.001), as illustrated in **Figures 4F** and **S7**. Additionally, we explored the potential relationship between A3s genes expression and stemness scores (RNAss and DNAss) and identified an inverse correlation between A3s expression and tumor stemness in the lymphoid neoplasm diffuse large B-cell lymphoma (DLBC), COAD, ESCA, GBM, and uterine carcinosarcoma (UCS). Noticeably, most A3s genes (except for A3A) exhibited positive correlations with DNAss and negative correlations with RNAss in LGG (**Figures S8A, B**).

### 3.5 Construction and validation of an A3s signature for predicting prognosis in glioma patients

**Figure 5:**
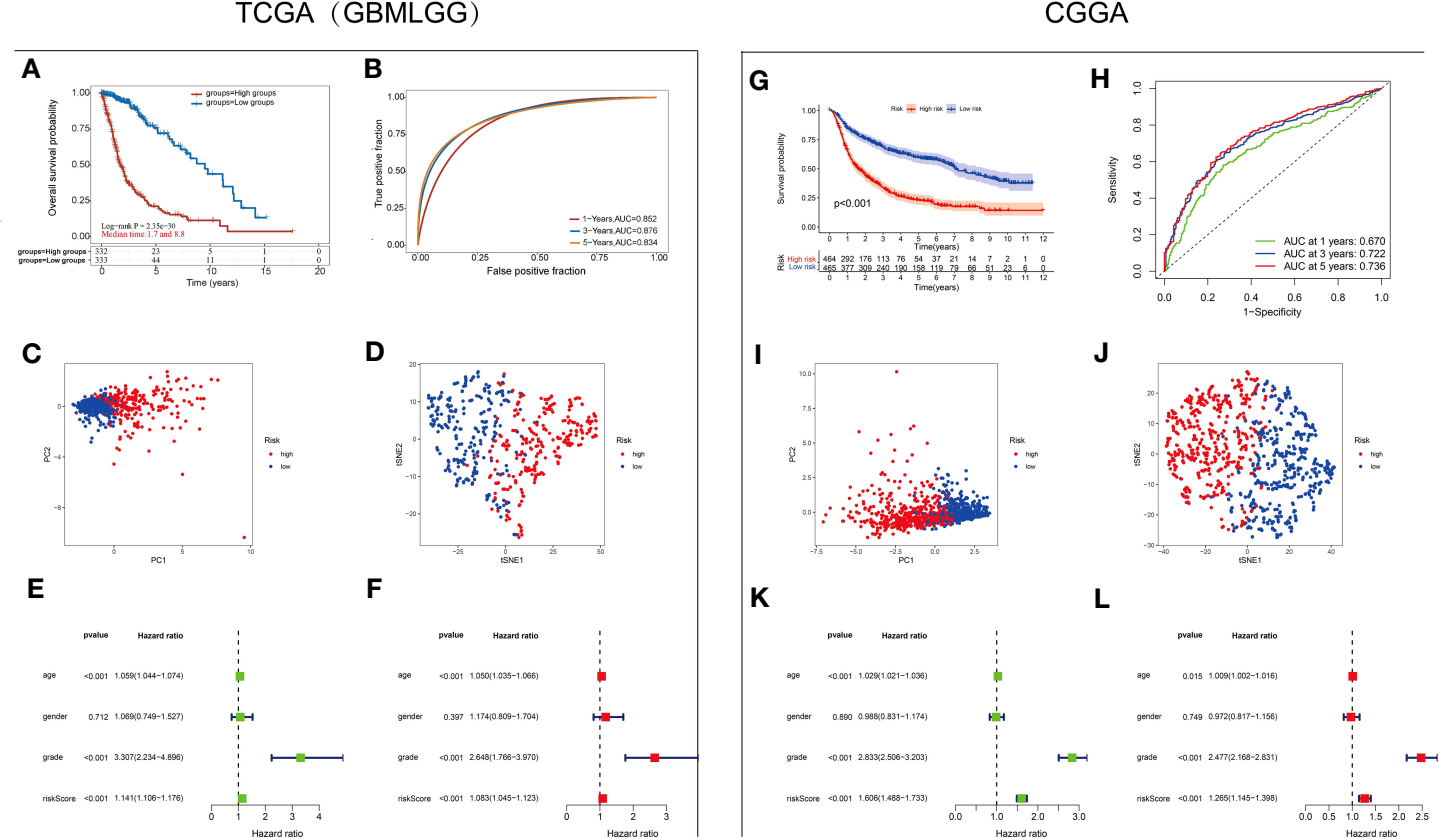
Establishment and validation of the A3s gene signature **(A)** Kaplan–Meier curves for the high-risk and low-risk groups in the TCGA-GBMLGG cohort. Log-rank test **(B)** ROC analysis of OS at 1, 3, and 5 years in the TCGA-GBMLGG cohort. **(C)** PCA and **(D)** dimensionality reduction by t-SNE of the high- and low-risk groups based on the risk score in the TCGA-GBMLGG cohort. Forest plot based on **(E)** univariate Cox regression analysis and **(F)** multivariate Cox regression analysis of the TCGA-GBMLGG cohort. **(G)** Kaplan–Meier curves for the high-risk and low-risk groups in the CGGA cohort. Log-rank test **(H)** ROC analysis of OS at 1, 3, and 5 years in the CGGA cohort. **(I)** PCA and **(J)** dimensionality reduction by t-SNE of the high- and low-risk groups based on the risk score in the CGGA cohort. Forest plots based on **(K)** univariate Cox regression analysis and **(L)** multivariate Cox regression analysis of the CGGA cohort.

A3 genes exhibited statistically significant P values and the highest HRs in glioma, indicating their potential as prognostic risk factors (**Figure 2**). Consequently, we aimed to determine the independent prognostic value of A3s expression in glioma. To this end, Cox regression analysis was employed to develop a predictive 7-gene signature using the TCGA and CGGA datasets. The following formula was derived: Risk score = (0.2715)*A3A + (0.0876)*A3B + (0.4262)*A3C + (0.2548)*A3D + (0.3729)*A3F + (0.215)*A3G + (-0.4655)*A3H. Furthermore, Kaplan–Meier analysis demonstrated that the high-risk group exhibited poorer OS than the low-risk group (**Figures 5A, G**). The areas under the receiver operating characteristic (ROC) curve for 1-, 3-, and 5-year OS were 0.852, 0.876, and 0.834, respectively, in the TCGA GBM/LGG cohort and 0.67, 0.722, and 0.736, respectively, in the CGGA cohort (**Figures 5B, H**), indicating the favorable predictive performance of the model (**Figures 5B, H**). These results underscore the predictive performance of the established signature. Moreover, principal component analysis (PCA) and t-distributed stochastic neighbor embedding (t-SNE) demonstrated distinct group separation (**Figures 5C, D, I, J**). Furthermore, univariate Cox regression analysis and multiple Cox regression analysis revealed that the risk score is an independent prognostic factor for OS in patients with glioma (**Figures 5E, F, K, L**).

### 3.6 Interaction and coexpression analyses of A3s

**Figure 6:**
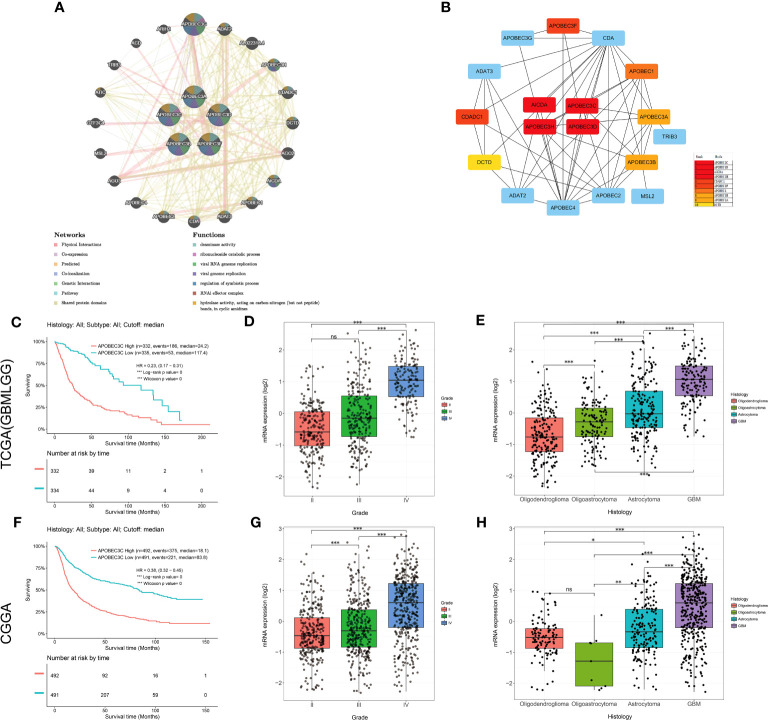
Upregulation of A3C expression is associated with the prognosis and clinicopathological characteristics of glioma. **(A)** The gene–gene interaction network for the APOBEC3 family was constructed using the GeneMANIA database **(B)** The top 10 genes were identified by sorting the 18 genes encoding these proteins with supporting data from hub gene analysis. **(C)** Kaplan−Meier survival curves of patients in the TCGA-GBMLGG cohort based on A3C expression. The clinical features related to A3C expression included **(D)** tumor grade and **(E)** histology, based on analysis of the TCGA-GBMLGG cohort. **(F)** Kaplan–Meier survival curves of patients in the CGGA cohort based on A3C expression. The clinical features related to A3C expression included **(G)** tumor grade and **(H)** histology, based on analysis of the CGGA cohort. *P< 0.05, **P< 0.01, ***P< 0.001, ns, no significance.

Then, the interaction network of A3s was mapped by using the GeneMANIA tool, revealing annotations pertaining to potential physical interactions, genetic interactions, coexpression, and shared pathways as well as protein domains. As shown in **Figure 6A**, A3s were mapped primarily to deaminase activity, ribonucleoside catabolism, regulation of symbiotic processes, RNAi effector complexes, hydrolase activity, acting on carbon-nitrogen (but not peptide) bonds in cyclic amidines, and viral genome/RNA replication pathways.Intriguingly, the utilization of the cytoHubba plugin facilitated the discovery of three hub A3s (A3C, A3D, and A3H), implying their potential importance within the network (**Figure 6B**).

### 3.7 Upregulation of A3C expression correlates with poor prognosis and unfavorable clinicopathological features in glioma

Among the aforementioned core genes, A3C exhibited the most substantial correlation with OS in glioma. We proceeded to conduct additional investigations to elucidate the association between A3C and clinical prognosis in glioma. Increased A3C expression was associated with poor prognosis, as well as high tumor grade and more unfavorable histology (TCGA dataset, P < 0.001, **Figures 6C–E**; CGGA dataset, P < 0.001, **Figures 6F–H**). To further validate these results, a thorough examination of the correlation between A3C protein expression and clinical-pathological characteristics of gliomas was undertaken. The results confirmed that A3C was expressed at higher levels in glioma tissues than in normal tissues (**Figure S9A**), and this upregulation was found to be associated with high tumor grade, unfavorable histology and activity in disease-related pathways in glioma (**Figures S9B–K**). Thus, aberrantly expressed A3C might regulate glioma progression and may be a therapeutic target for glioma.

### 3.8 Functional enrichment analysis of A3C-related partners in glioma

To gain deeper insights into the functional attributes of the A3C gene within the context of glioma, we identified genes correlated with A3C. Subsequently, the top 300 correlated genes were subjected to KEGG and GO enrichment analyses. The identified KEGG pathways encompassed crucial processes such as PD-L1 expression, PD-1 checkpoint pathway in cancer, cell adhesion molecules (CAMs), viral myocarditis, p53 signaling pathway, NF-kappa B signaling pathway, human T-cell leukemia virus 1 infection, human immunodeficiency virus 1 infection, toll-like receptor signaling pathway, and phagosome, all of which might be involved in the effect of A3C on tumor pathogenesis (**Figure S10A**). The results of the GO enrichment analysis demonstrated significant associations between most of these genes and various pathways and cellular processes related to the immune responses, immune system function, cell activation, vesicle dynamics, leukocyte activation, immune effector processes, myeloid leukocyte activation, cytoplasmic vesicles, and intracellular vesicles (**Figures S10B–D**), further supporting the potential role of A3C in glioma progression through these processes.

### 3.9 Upregulation of A3C expression is correlated with immune infiltration in glioma

**Figure 7:**
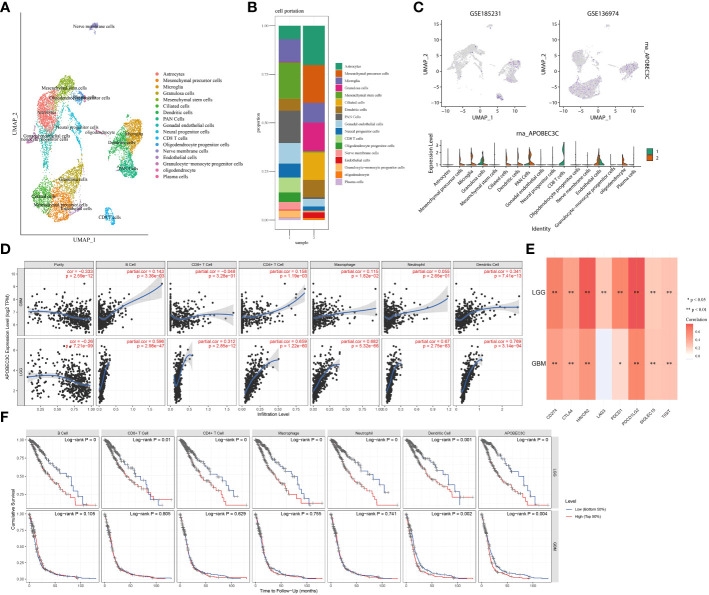
Relationships between A3C expression and glioma cell subpopulations. **(A–C)** A3C was expressed mainly in astrocytes, CD8+ T cells, and microglia in glioblastoma. **(D)** Associations between A3C expression and immune infiltration in GBM and LGG. **(E)** Associations between A3C expression and immune checkpoint expression in GBM and LGG. **(F)** Prognostic associations between A3C expression and immune infiltration in GBM and LGG. *P<0.05, **P< 0.01.

To provide additional evidence of the association between A3C expression and immune infiltration, we analyzed single-cell RNA-seq datasets (GSE185231 and GSE136974). Our findings revealed upregulated A3C expression within malignant GBM, specifically in CD8+ T cells, microglia, and endothelial cells (**Figures 7A–C**). We further utilized the TCGA database to substantiate the connection between A3C expression and immune cells across diverse glioma grades. Interestingly, the correlations between A3C expression and immune cell infiltration varied across different grades of glioma (**Figure 7D**) Then, we continued to explore the correlations between A3C expression and immune checkpoint expression and found that in comparison to GBM, LGG exhibited elevated immune cell infiltration and increased expression of immune markers, such as PD-L1, indicating a more favorable immune response within the TME. Indeed, the expression of A3C displayed positive correlations with CD274, PDCD1, PDCD1LG2, SIGLEC15, CTLA4, HAVCR2, and GZMB in glioma (P < 0.05; **Figure 6D**). The correlations between A3C expression and the expression of the immune checkpoint genes CD274, HAVCR2, PDCD1, LAG3, and PDCD1LG2 were significantly higher in LGG tumors than in GBM tumors (**Figure 7E**). Additionally, a correlation was observed between A3C expression in immune cells and survival in glioma patients, with this metric demonstrating significant prognostic value in LGG compared to GBM (**Figure 7F**). We hypothesize that the interaction between elevated A3C and the immune system might harbor the potential to influence the malignant progression of glioma.

### 3.10 A3C is correlated with stemness in glioma

**Figure 8:**
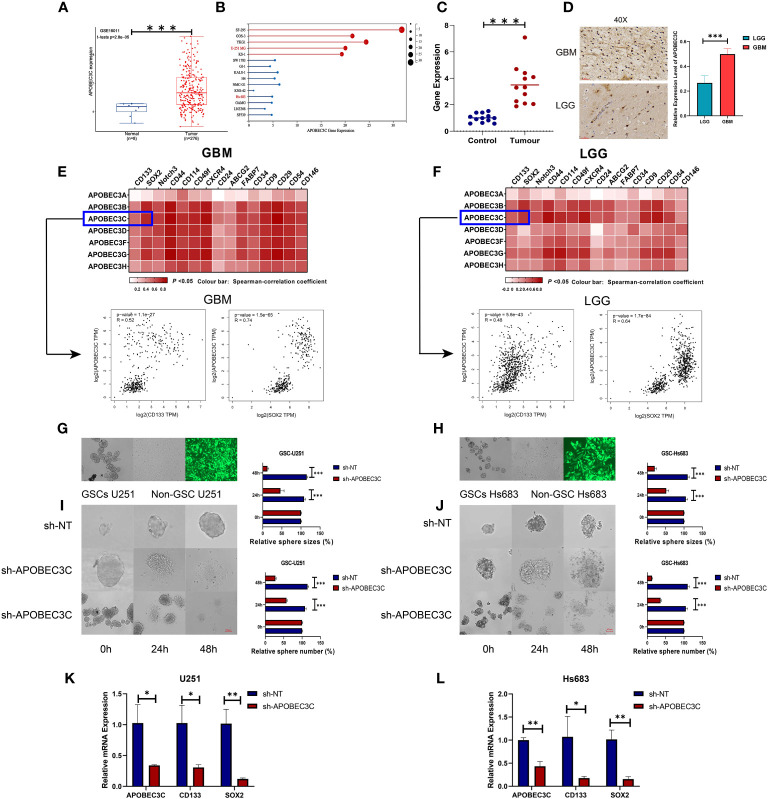
A3C is significantly upregulated in glioma and is involved in maintenance of self-renewal in glial stem cells. **(A)** Boxplot showing A3C expression in different glioma tissues in the GEO database. **(B)** Expression level of A3C in different glioma cell lines (data from CCLE). **(C)** RT–qPCR results showing increased serum levels of A3C in GBM patients. **(D)** Evaluation of A3C expression by immunohistochemical staining in glioma patients. **(E, F)** GEPIA was used to show the correlations between A3 expression and stemness gene expression in GBM and LGG patients. **(G, H)** Representative images of neurosphere-cultured glial stem cells and two established glial stem cell lines. **(I, J)** Sphere formation of glioma cells was evaluated by the sphere formation assay. Knockdown of A3C inhibited sphere formation in glioma stem cells. T. Bar: 100 μm. (K,L) Knockdown of A3C inhibited stem cell marker expression by RT-qPCR. *P< 0.05, **P< 0.01, ***P< 0.001.

To confirm the elevation of A3C in glioma tissues and cells and explore its possible correlation with glioma progression, we analyzed external datasets and performed *in vitro* experiments (**Figure 8**). First, the upregulation of A3C in glioma tissues was determined through analysis of the GSE16011 dataset and found to be statistically significant (P< 0.001, **Figure 8A**). Furthermore, the expression levels of A3C were examined in GBM (n=5) and LGG (n=10) cell lines, with significantly higher levels observed in GBM cells than in LGG cells (**Figure 8B**). Additionally, A3C expression was assessed by RT–qPCR in whole blood samples obtained from a cohort of 12 GBM patients and 12 healthy controls, with significantly higher expression found in the GBM patients than in the controls (**Figure 8C**). Subsequently, analysis of six archived clinical glioma specimens (three cases each of LGG and GBM) was performed. We found significantly higher levels of A3C expression in GBM (AOD=0.4985) than in LGG (AOD=0.26575), as shown in **Figure 8D**.

The importance of stem-like cell properties in cancer initiation, progression, and metastasis cannot be understated. We assessed commonly expressed stem cell markers in both LGG and GBM. The results indicated a stronger correlation between A3s expression and the presence of common stem cell markers in GBM compared to LGG, as displayed in **Figures 8E, F**. This finding suggests a positive association between A3s expression and the extent of glioma stemness, alongside the tumor grades. To further explore the role of A3C, *in vitro* experiments were conducted using GSC-U251 and GSC-Hs683 cell lines. A3C expression was decreased using lentiviral vectors containing shRNA-APOBEC3C, with an infection efficiency of nearly 100% in both cell lines after 72 h (**Figures 8G, H**). Knockdown of A3C inhibited sphere formation in glioma stem cells (**Figures 8I, J**). Following A3C knockdown, the expression of tumor stemness markers (CD133, SOX2), exhibiting a strong correlation with A3C expression, was subsequently diminished compared to the control (**Figures 8K, L**). These results suggest a potential role for A3C in maintaining glial stemness.

### 3.11 Inhibition of glioma cell proliferation, migration, and invasion in glioma cells following A3C knockdown

**Figure 9:**
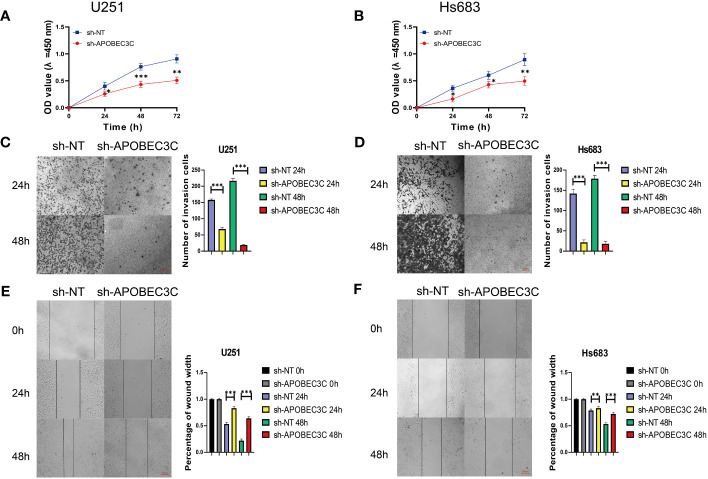
A3C knockdown impairs glioma cell proliferation, migration, and invasion. **(A, B)** Cell viability was tested by CCK8 assay. **(C, D)** Cell invasion was assessed by transwell migration invasion assays. **(E, F)** Cell migration was evaluated by wound healing assays. *P< 0.05, **P< 0.01, ***P< 0.001.

In glioma cells, knockdown of A3C was accompanied by significantly decreased proliferation and the induction of apoptosis in U251 and Hs683 cells compared to the negative control cells (P < 0.05), as shown in **Figures 9A, B**. Additionally, A3C knockdown resulted in decreased invasion of both U251 and Hs683 cells (P < 0.05; **Figures 9C, D**). Furthermore, the wound healing assay revealed that control cells exhibited a higher migration rate than U251 and Hs683 cells lentivirally transduced with sh-A3C (P < 0.01; **Figures 9E, F**). These findings suggest that inhibition of A3C expression leads to reductions in the proliferation, invasion, and migration of glioma cells.

## 4 Discussion

Recent research has shed light on the significant role of A3 enzymes in the molecular pathogenesis of cancer, primarily through the deamination of cytidine to uridine in DNA and RNA. This enzymatic activity is intricately involved in regulating diverse biological processes, including protein expression and innate immunity (33). Research on this enzyme family has predominantly focused on their antiviral function in innate immunity and retrospective analyses of tumor mutations stemming from aberrant DNA editing mechanisms and promiscuous RNA editing, strongly associated with cancer development (34–36). A3s have demonstrated robust inhibitory effects on HBV and have been extensively documented for their involvement in the initiation and progression of various human malignancies (37, 38). Notably, induction of non-replicative chromosomal instability by A3A could promote pancreatic cancer (39) metastasis, while its association with human papillomavirus-linked oropharyngeal cancer (40) involved viral genome integration. Besides, A3B’s influence on the tumor microenvironment was found to contribute to hepatocellular carcinoma progression (41). Aberrant A3B expression in breast cancer has been correlated with proliferation and cell cycle stage modulation (42). The complete deletion of homozygous A3B has emerged as a favorable independent prognostic factor for male LGG patients (38). Additionally, dysregulation of A3G triggers DNA damage and genomic instability in multiple myeloma (43). Moreover, it plays a pivotal role in facilitating oncogenic transformation and clonal evolution in bladder cancer and promoting liver metastasis in colorectal cancer (44, 45). Besides, A3A, A3D, and A3G have been linked to poor prognosis in renal cell carcinoma (46–48). However, the precise role of A3 enzymes in tumor development remains controversial. Accordingly, we conducted a comprehensive bioinformatics analysis to better understand A3s concerning their expression, genetic alterations, survival prognostics, and immune infiltration across a spectrum of cancer types. The distinct expression patterns of A3s were observed in various cancer types and cells. Survival analysis revealed all seven A3 genes as risk factors in the GBMLGG. Current molecular biomarkers exhibit limited ability in predicting glioma progression and associated risks. Thus, the quest for novel molecular markers holds great significance in enhancing prognosis and immunotherapeutic strategies for glioma. Given the substantial alterations in A3 gene expression and the finding that A3s genes exhibit statistically significant upregulation and the highest HRs in glioma, Cox regression analysis was employed to develop a predictive 7-gene signature. Univariate and multivariate Cox regression analyses revealed that the risk score is an independent prognostic factor for OS in patients with glioma.

In recent years, immunotherapy strategies have experienced a growing utilization in the management of various types of cancers (49), including brain cancers (50). As indicated by parameters like TMB and MSI, the genomic heterogeneity of tumors greatly influences treatment responses to immune checkpoint inhibitors and prognosis (51). DNA methylation and MMR activate tumor suppressors and inactivate oncogenes (52, 53). Our study revealed a positive relationship between A3s expression and methylation and MMR patterns in several cancers, implying A3s’ potential influence through DNA methylation and MMR in cancers like LGG and LUAD. Elevated TMB and MSI are often observed in certain cancer types and affect patient survival (54). High TMB has been reported to predict positive immune-checkpoint inhibitor responses and prolongs overall survival (31, 55), while MSI could predict tumor genesis and progression (56). The present study established significant positive associations between A3s expression and TMB/MSI in BLCA, BRCA, and COAD, whereas negative associations were noted in ESCA, PAAD, PCPG, and TGCT. Consequently, A3s expression may predict immunotherapy efficacy, especially in tumors exhibiting high MSI and TMB. Our results highlight the correlation between A3s and the tumor microenvironment in human cancers, potentially making A3s valuable tumor biomarkers for immune treatment.

The central nervous system has a unique immune environment; however, it can still mount an effective antitumor immune response. Therefore, immunotherapy has great therapeutic potential for gliomas. GBM frequently exhibits significant immunosuppression, characterized by an increase in immunosuppressive cell populations and the activation of immune evasion mechanisms. These features are accompanied by increased invasiveness and malignancy, leading to a relatively unfavorable response to treatment. In contrast, LGG may demonstrate enhanced immune responsiveness. LGGs tend to grow more slowly and may display greater susceptibility to treatment (57). We found that the correlations between A3C expression and the expression of the immune checkpoint genes were significantly higher in LGG tumors than in GBM tumors. Overall, the contrasting biological and clinical contexts of GBM and LGG could impact the correlation between A3C genes and immune markers. Previous studies have suggested that A3 enzymes inhibit retroviral infections and retrotranspositions (58, 59), with their expression activated in response to immune signals like interferon-alpha (60). A3A was discovered to harbor catalytic activity that can cause DNA damage during replication, leading to PD-L1 upregulation (61). Besides, A3B triggers immune responses in cancers by influencing neoepitopes, thereby enhancing immunotherapy outcomes (62). More remarkably, among the A3s, A3C was identified as the hub gene and had the highest correlation with OS in glioma. We thus further investigated A3C in glioma. In our analyses, utilizing an A3C-related gene subset in glioma, KEGG pathway enrichment and GO enrichment analyses revealed significant enrichment in multiple cancers, inflammation-related pathways, apoptosis, migration/invasion pathways, and regulation of immune-related biological processes and pathways. This leads us to speculate that A3C might influence tumor progression by interacting with the aforementioned signaling pathways.

Several factors that contribute to the complexity of treating GBM. However, the three primary factors of significance are GSCs, tumor heterogeneity, and the blood-brain barrier (63). Specifically, GSCs play a crucial role in tumor heterogeneity, as well as in the development of radio-resistance, chemo-resistance, and recurrence in GBM (64). In the glioma microenvironment, GSCs secrete cytokines that promote tumor invasion, immune cell recruitment, angiogenesis, and self-renewal, thereby contributing to cancer progression and recurrence (65). However, the regulatory mechanisms governing glial stem cells and their acquisition of invasive and migratory traits remain incompletely understood. Importantly, we found a positive correlation between A3C and glioma stem cell marker genes such as CD133, SOX2, Notch3, CD44, and CXCR4 was noted. Though cell experiments, we found that the depletion of A3C leads to the downregulation of markers of glioma stem cells (CD133 and SOX2) and a decrease in stemness, underscoring its potential importance in maintaining glial stemness. Furthermore, enrichment analysis of A3C-related partners in glioma highlighted significant correlations with activating or inhibiting various carcinogenic pathways and immune functions. In addition, we also found that A3C and A3G show high expression correlation, similar immune cell profiles, and correlation with stemness marker expression in GBM and LGG. A3G knockdown not only attenuates proliferation, invasion in glioma-initiating cells but also inhibits the initiation of glioma spheres (66). We elevated A3G expression was significantly associated with unfavorable prognosis as well as clinical pathology of glioma through the utilization of the TCGA-GBMLGG and CGGA dataset (**Supplementary Figures 11A–F**) and confirmed a correlation between the expression of A3G and A3C (**Supplementary Figures 11G, H**). Moreover, Interestingly, knockdown of A3C resulted in downregulation of A3G expression in U251 and SH683 cells (**Supplementary Figure 11I**)ure. Given the above results, A3C might regulate cancer progression by interacting with A3G. However, our study represents a starting point in investigating A3C’s function in cancer stem cells and immune functions; the underlying pathways require further elucidation, and additional research is warranted to obtain a better understanding.

Despite our comprehensive analysis, several limitations are noteworthy. Functional verification experiments were conducted exclusively in cultured cells, and using microarrays from diverse public datasets may introduce systematic bias. Consequently, the precise mechanisms through which A3s participate in immune and stemness regulation remain incompletely understood and warrant further exploration. A refined understanding of A3s’ roles in cancer immune infiltration is vital for the development of effective antitumor immunotherapy drugs targeting A3s. The A3 family holds pivotal roles across multiple cancer types. A3s can potentially facilitate glioma development, with A3C impacting the tumor microenvironment and stem cell characteristics. These discoveries provide significant insights into the clinical significance of A3s in cancer and offer guidance for potential therapeutic interventions.

## Data availability statement

The original contributions presented in the study are included in the article/**Supplementary Material**. Further inquiries can be directed to the corresponding authors.

## Ethics statement

The studies involving humans were approved by Ethics Committee of Nanyang Central Hospital. The studies were conducted in accordance with the local legislation and institutional requirements. The participants provided their written informed consent to participate in this study.

## Author contributions

QX and ZQ conceived and designed the study. SZ, FB, and YG collected the data. YG, KH, and SZ analyzed and interpreted the data. XS and ZW collected samples. YH, XG, and QD verified the data. SZ and YG performed all statistical analyses. QX, ZQ, and HZ wrote the manuscript. All authors contributed to the article and approved the submitted version.
