# Supplementary material for: The role of APOBEC3C in modulating the tumor microenvironment and stemness properties of glioma: evidence from pancancer analysis
Source: Front Immunol. 2023 Sep 21;14:1242972. doi: 10.3389/fimmu.2023.1242972 (PMC10551170; doi:10.3389/fimmu.2023.1242972)
Supplement: Supplementary file 1 [file DataSheet_1.docx]

Supplementary Material

## Supplementary Figures

**
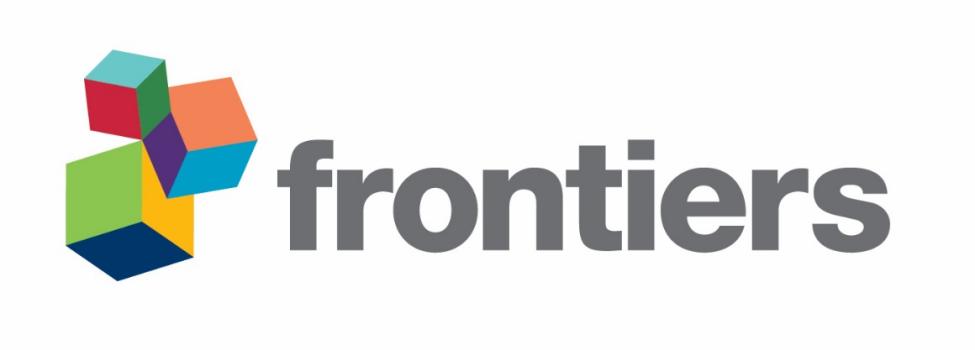
**


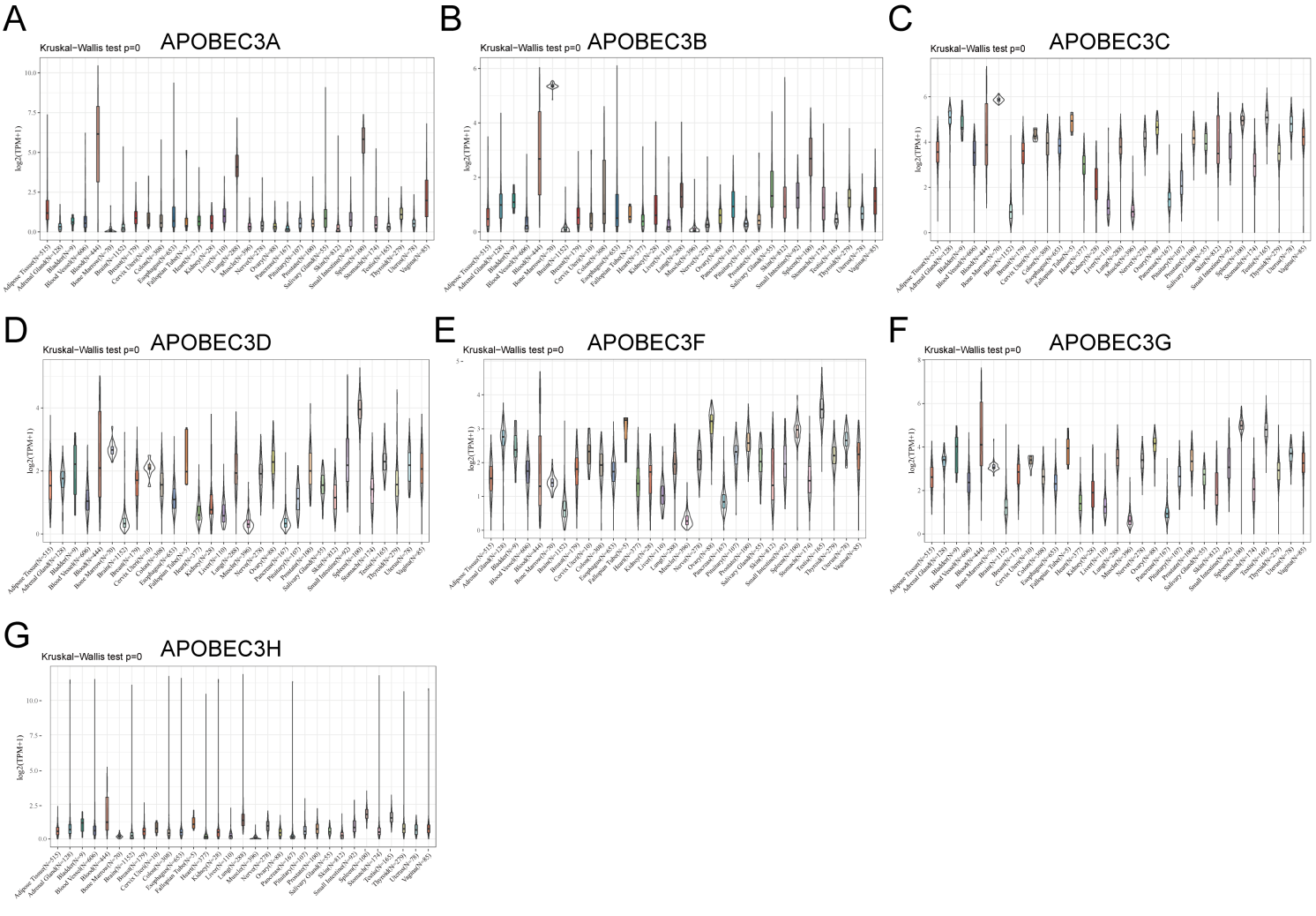


## Supplementary Figure 1. A3s expression patterns in diverse healthy tissues. Expression of (A) A3A, (B) A3B, (C) A3C, (D) A3D, (E) A3F, (F) A3G, and (G) A3H. Data from GTEx.

##
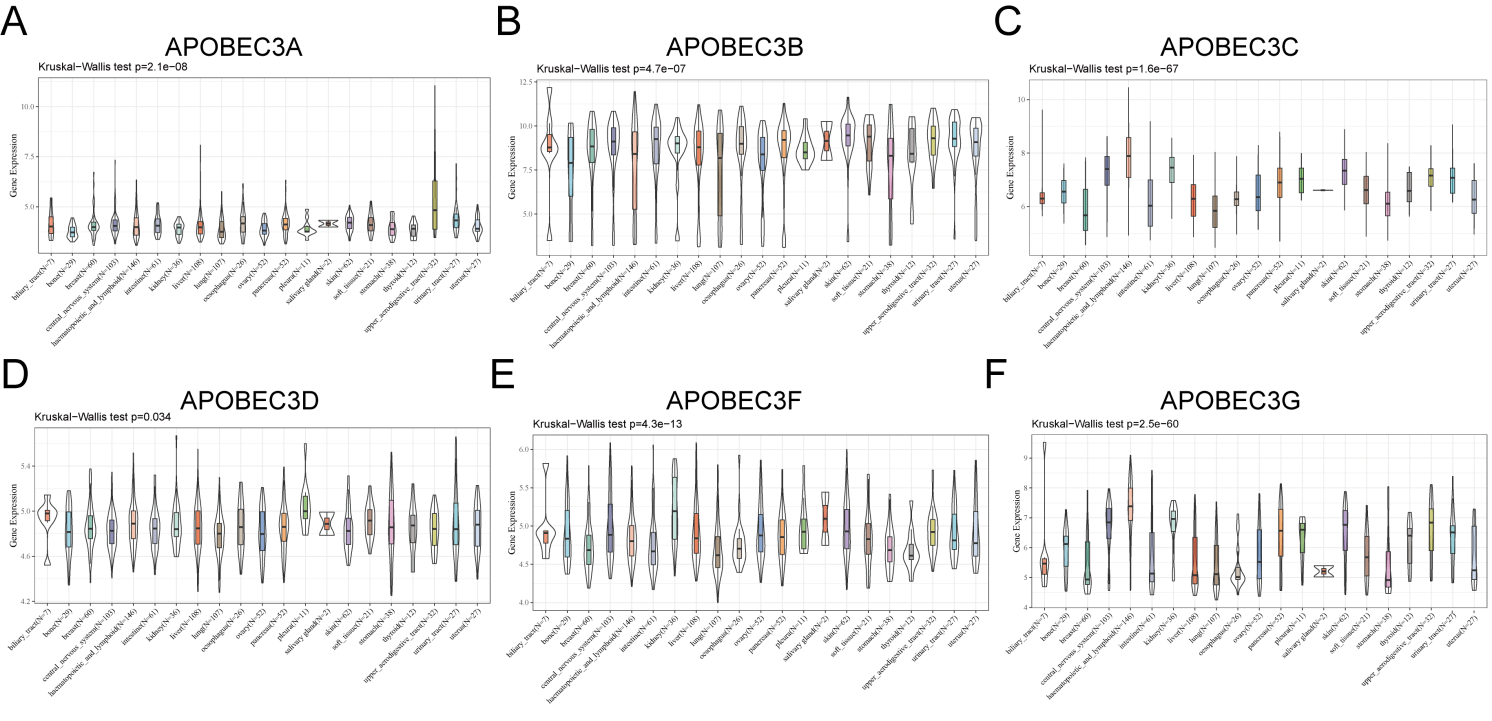
Supplementary Figure 2. A3s expression patterns in diverse cancer cell lines. Expression of (A) A3A, (B) A3B, (C) A3C, (D) A3D, (E) A3F, (F) A3G, and (G) A3H. Data from CCLE.

##
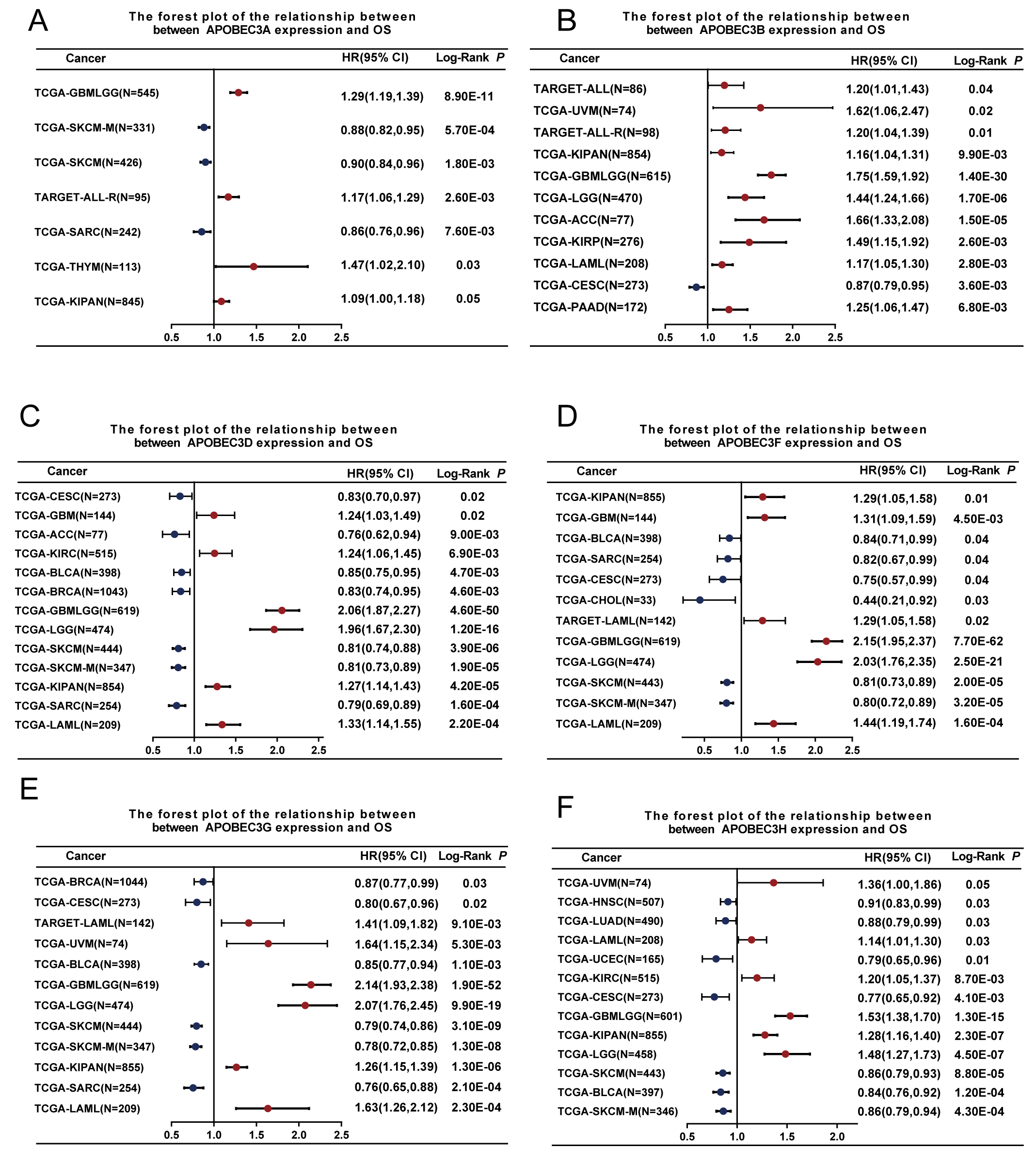
Supplementary Figure 3. Prognostic relevance of A3s.The relationship between (A) A3A, (B) A3B, (C) A3D, (D) A3F, (E) A3G, and (F) A3H expression and overall survival.

##
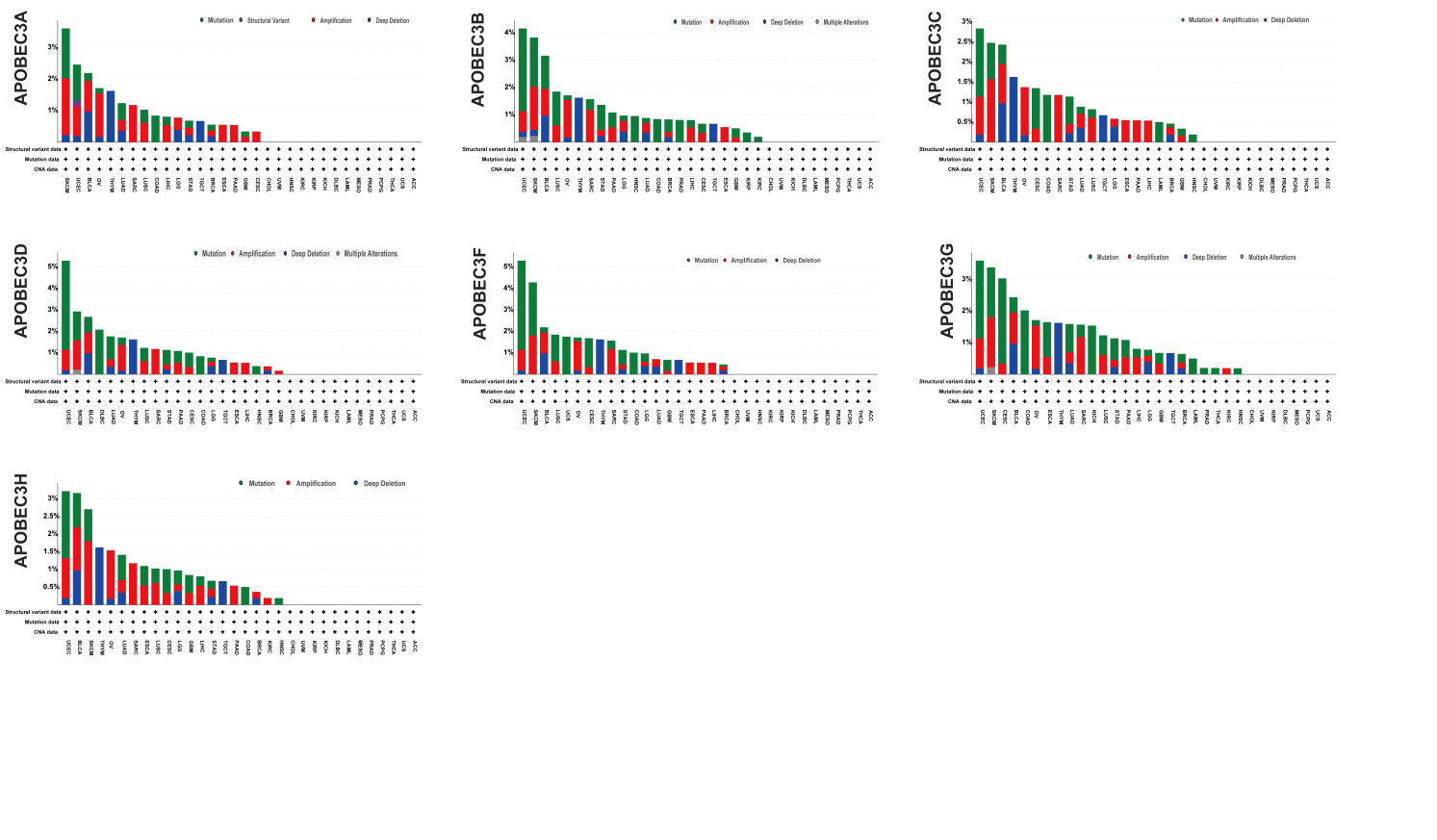
Supplementary Figure 4. Analysis of genetic alterations of each A3s genes in different tumors of TCGA.

##
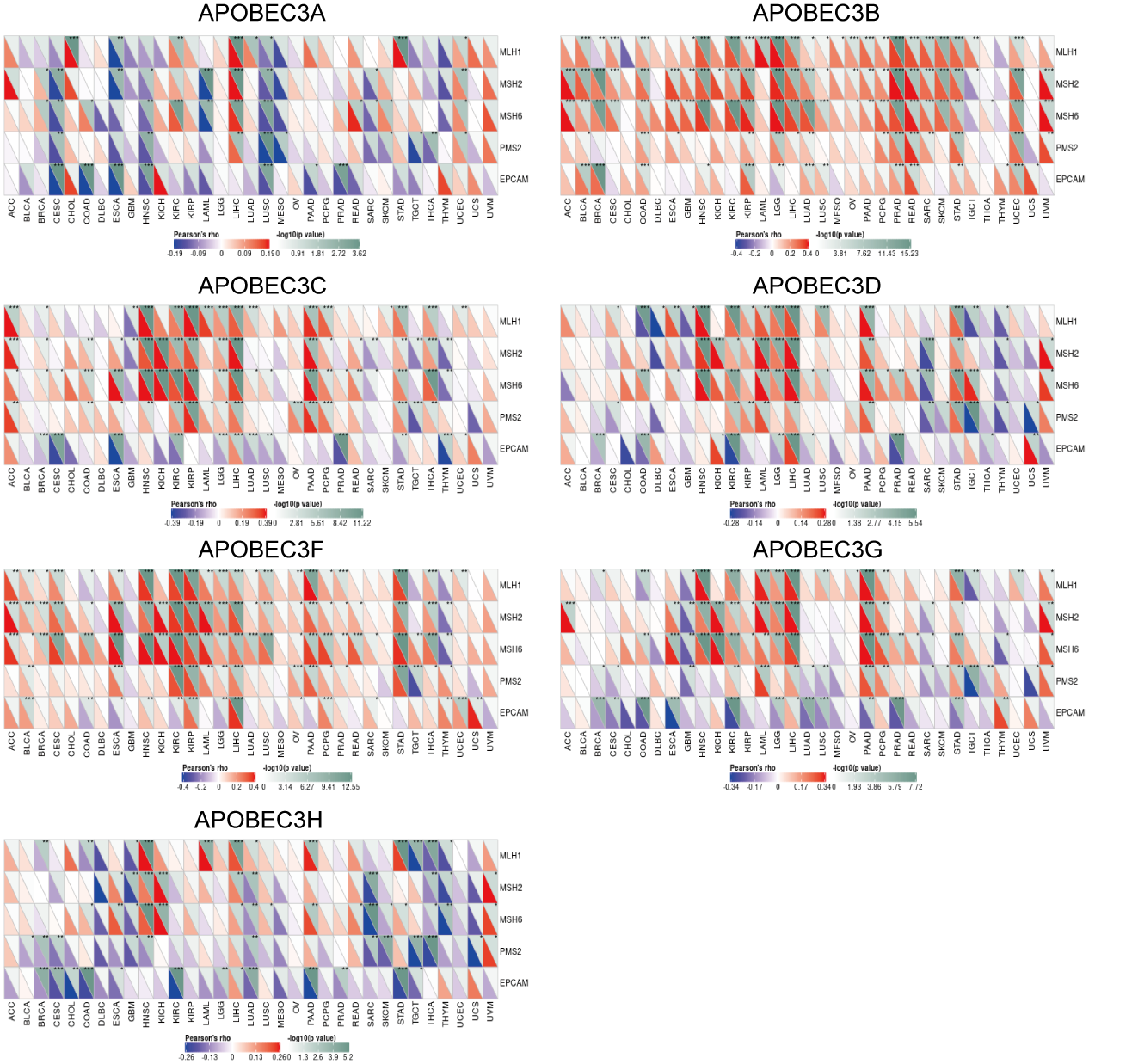
Supplementary Figure 5. Correlation between expression of A3s and MMR genes. *P< 0.05, ** P< 0.01, *** P< 0.001.

##
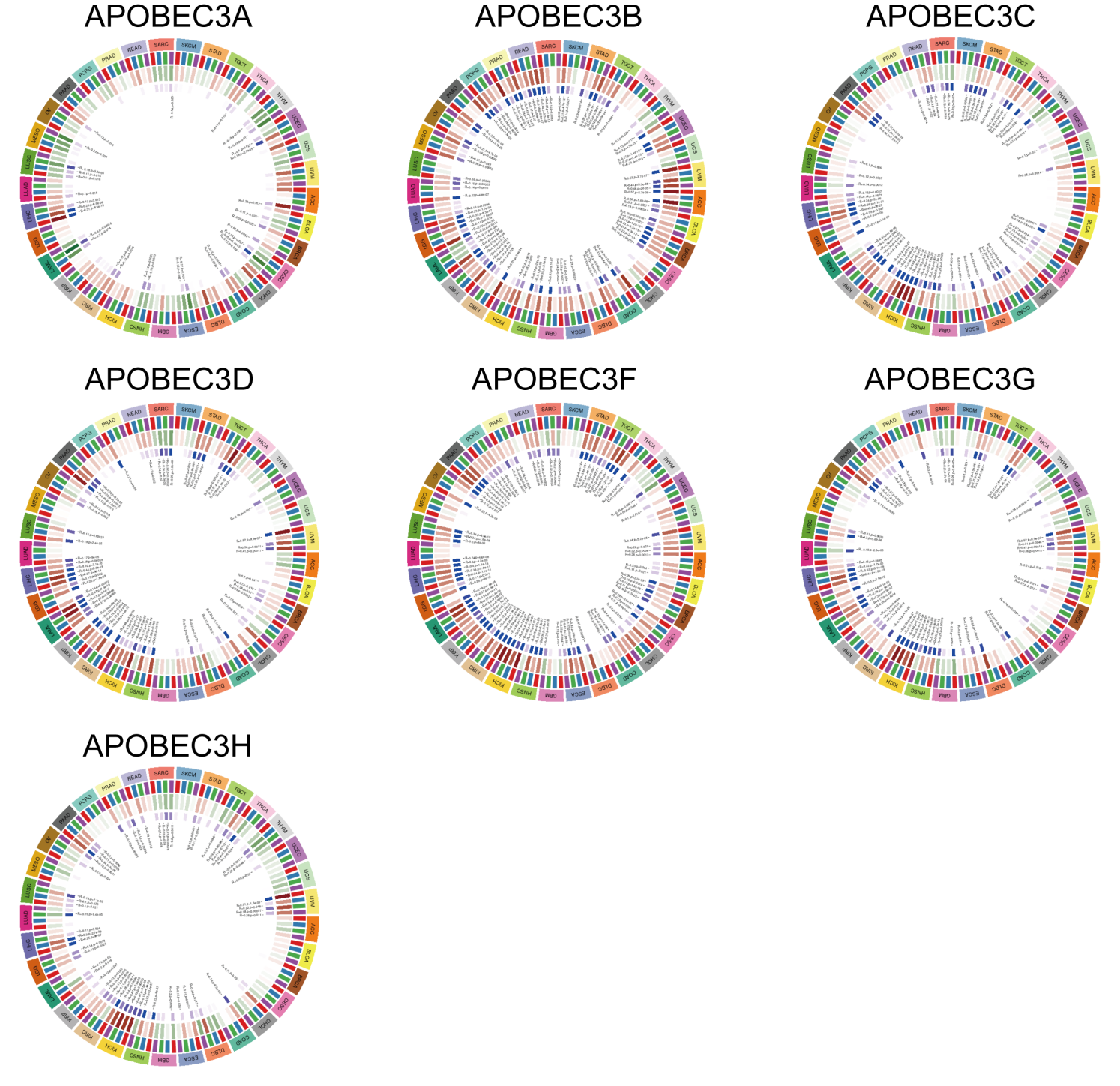
Supplementary Figure 6. Correlation between the expression of A3s and DNA methyltransferases. *P< 0.05, **P< 0.01, ***P< 0.001.

##
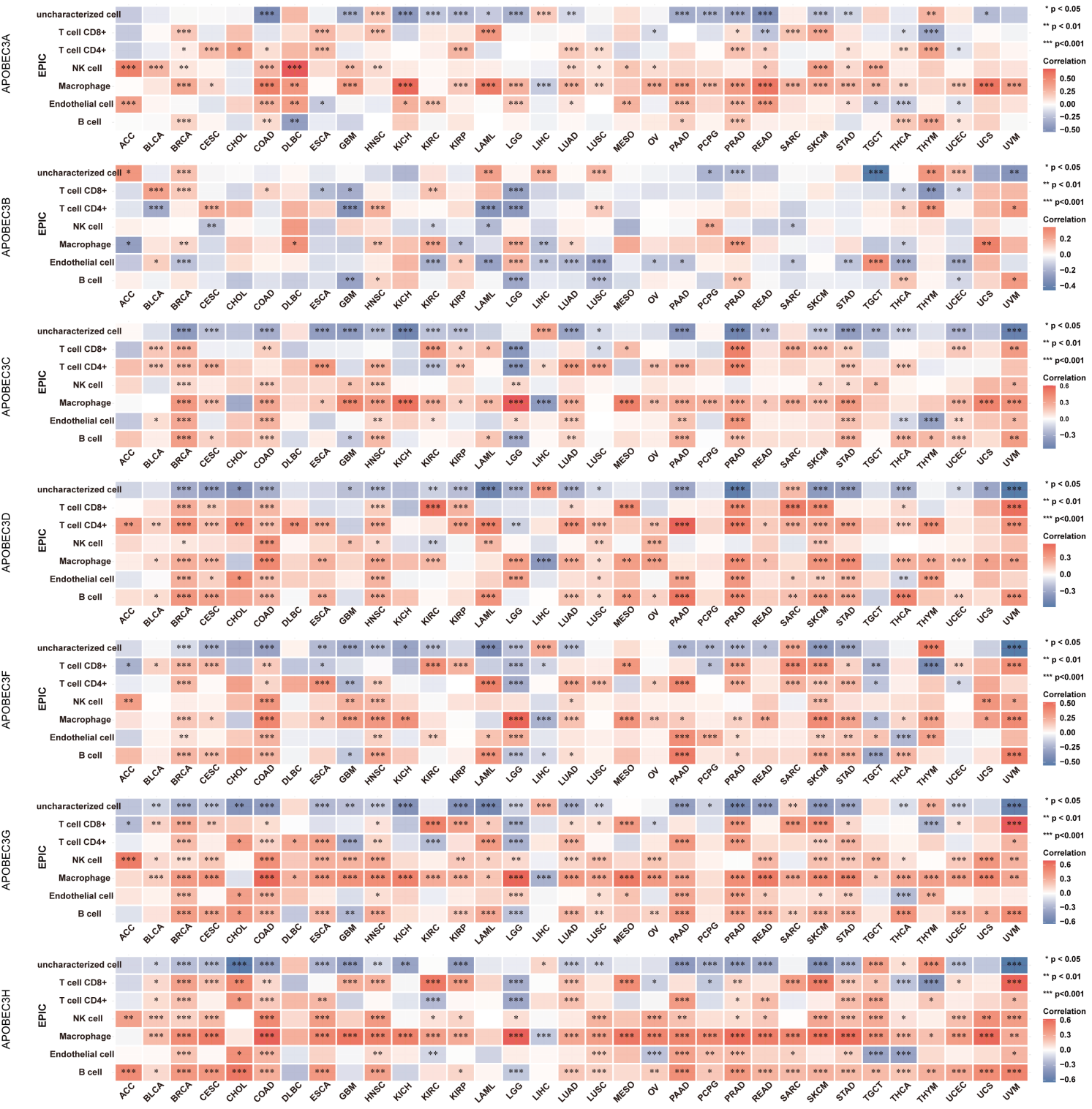
Supplementary Figure 7. Spearman correlation analysis heatmap of A3s expression and EPIC immune infiltration score in multiple tumor tissues. *P<0.05, **P< 0.01, ***P< 0.001.

##
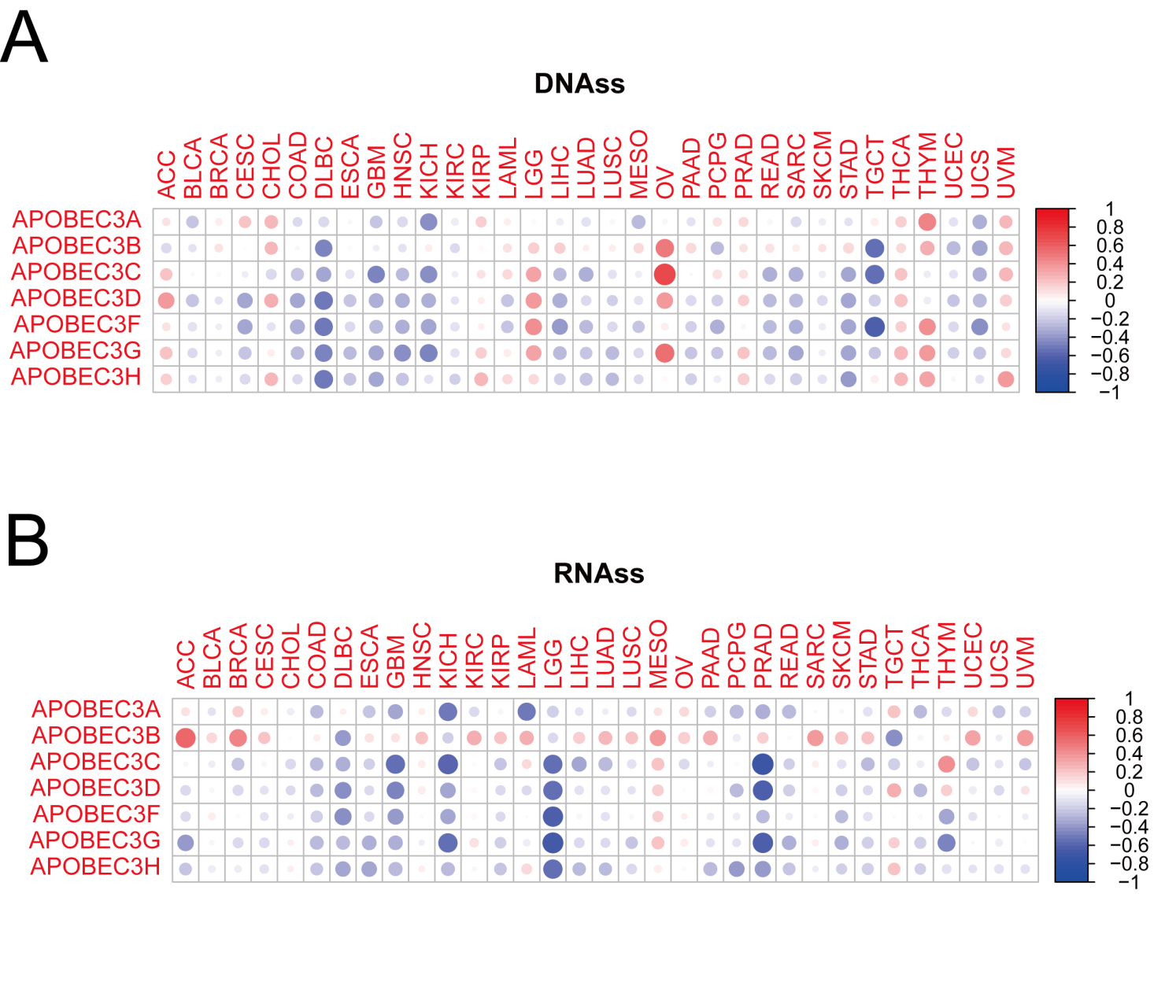
Supplementary Figure 8. Correlation between A3s expression and tumor stemness. Correlation matrix of A3s expression and cancer (A) RNAss and (B) DNAss, based on Spearman correlation tests.


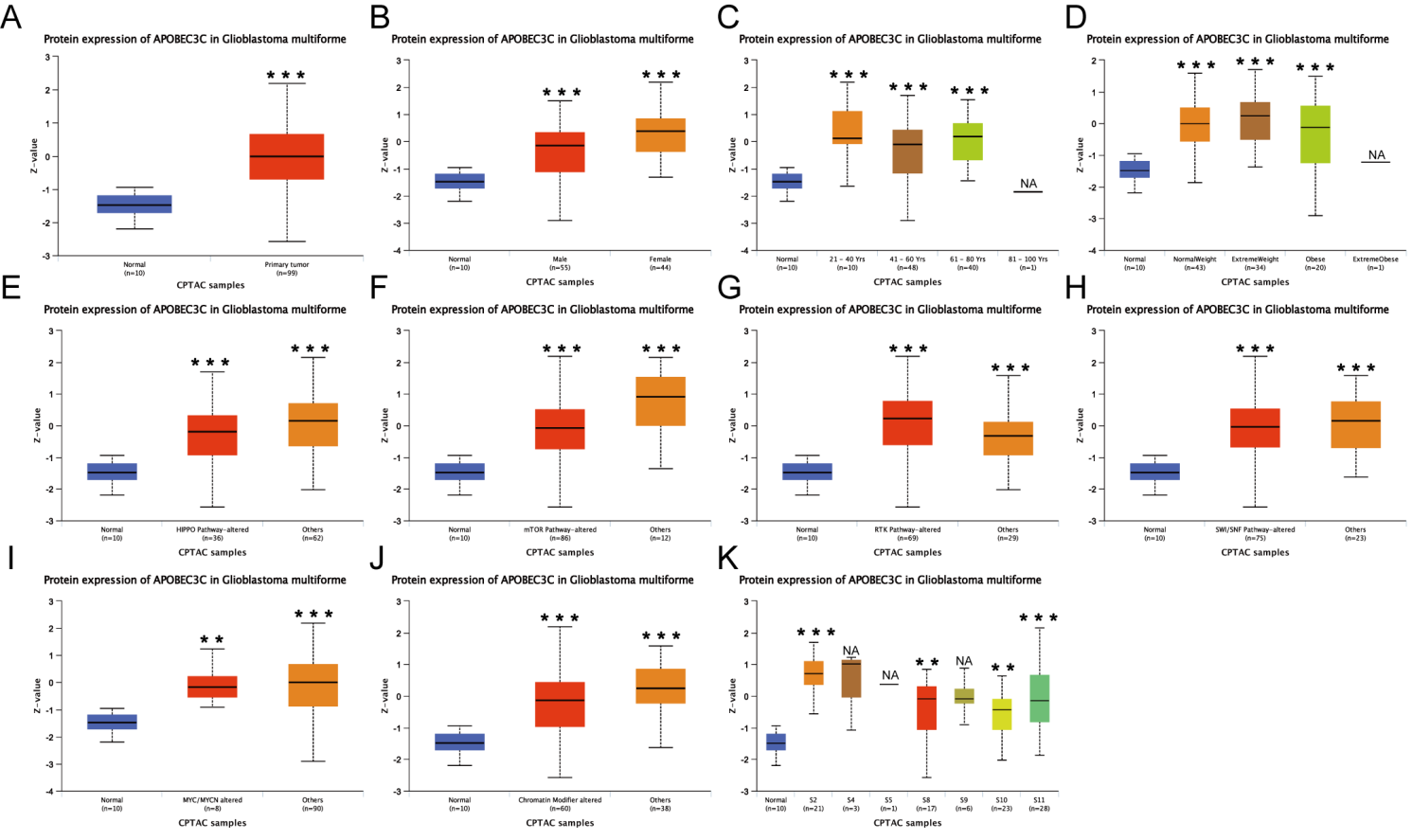
Supplementary Figure 9: The relationship between A3C protein expression and pathological characteristics of patients with glioma.*P <0.05, **P < 0.01, ***P < 0.001.

**
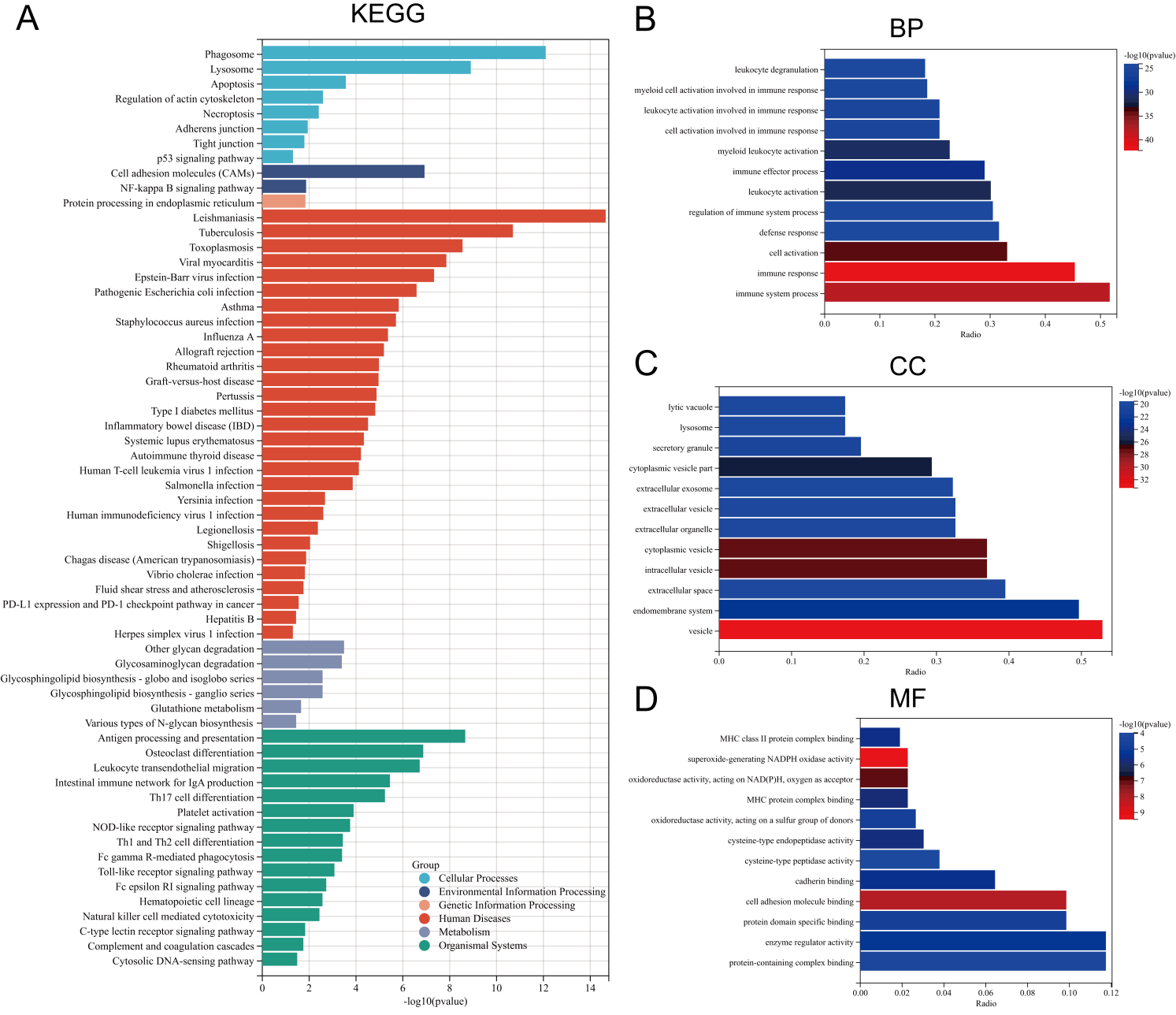
Supplementary Figure 10:**The KEGG and GO analyses results of the top 300 A3C-correlated targeting genes. (A) KEGG (B) GO: biological process; (C) GO: cellular component; (D) GO: molecular functions.


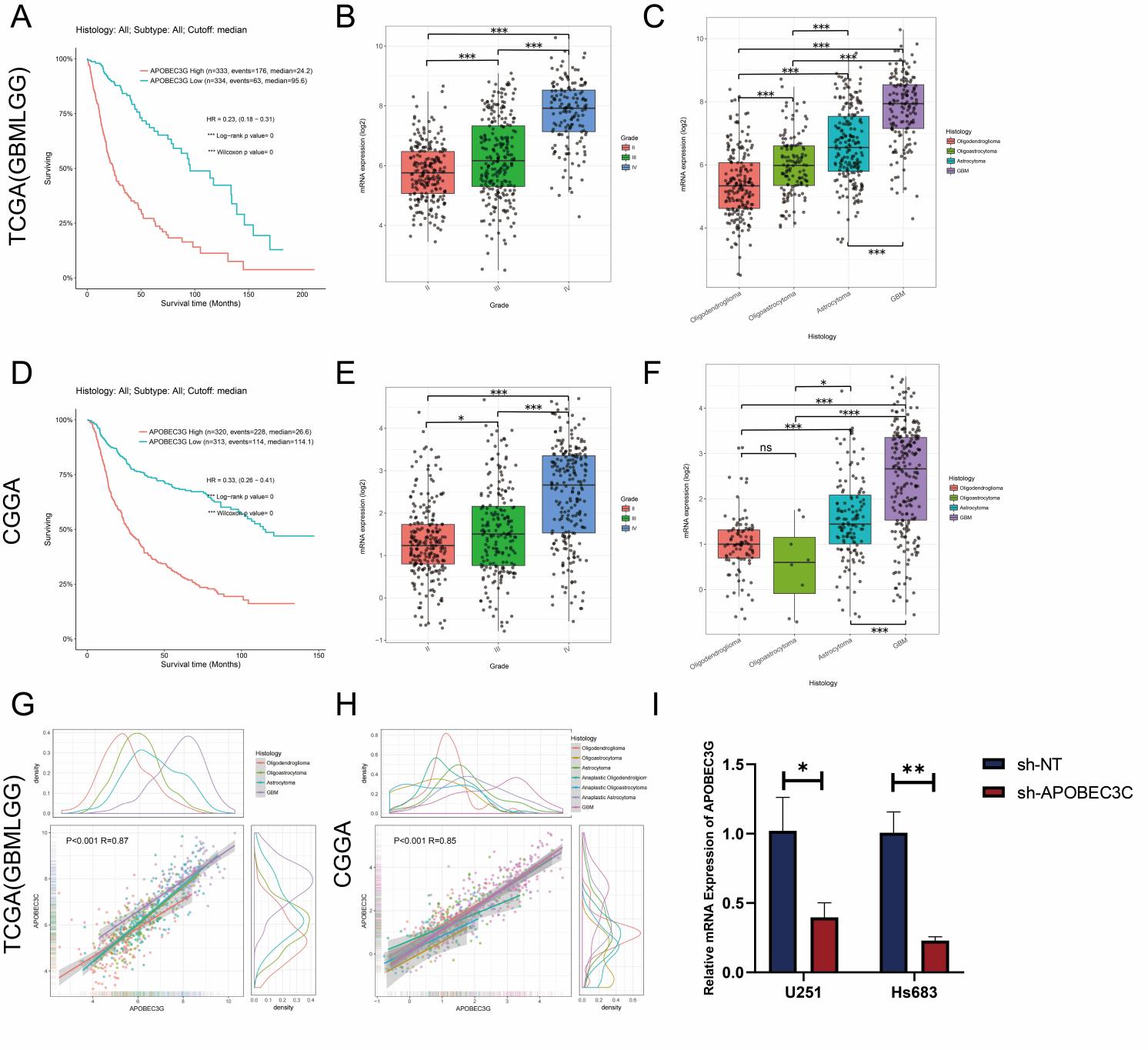
**Supplementary Figure 11:**Upregulation of A3G expression is associated with the prognosis and clinicopathological characteristics of glioma. (A) Kaplan-Meier survival curves of APOBEC3C based on TCGA-GBMLGG dataset.(B-C)The different expression of APOBEC3G in clinical features included (B) Grade and (C) Histology based on TCGA-GBMLGG dataset. (D) Kaplan-Meier survival curves of APOBEC3G based on CGGA dataset. (E-F)The different expression of APOBEC3C in clinical features included (E) Grade and (F) Histology based on CGGA dataset.(G) (G-H) Correlation between the expression of A3C and A3G based on (G)TCGA-GBMLGG dataset and (H) CGGA dataset. (I)A3C knockdown resulted in downregulation of A3G expression in U251 and Hs683 cells.

## Supplementary **Table**

**Table S1:**List of the top 300 genes that associated with A3C.

| Gene Symbol | Gene ID | PCC |
| --- | --- | --- |
| APOBEC3G | ENSG00000239713.7 | 0.84 |
| CASP8 | ENSG00000064012.21 | 0.83 |
| MR1 | ENSG00000153029.14 | 0.81 |
| S100A11 | ENSG00000163191.5 | 0.81 |
| APOBEC3F | ENSG00000128394.16 | 0.81 |
| ADPRH | ENSG00000144843.11 | 0.8 |
| NAGA | ENSG00000198951.11 | 0.8 |
| RNF135 | ENSG00000181481.13 | 0.79 |
| HFE | ENSG00000010704.18 | 0.79 |
| ARPC1B | ENSG00000130429.12 | 0.78 |
| SQRDL | ENSG00000137767.13 | 0.78 |
| CLIC1 | ENSG00000213719.8 | 0.78 |
| GAL3ST4 | ENSG00000197093.10 | 0.77 |
| CASP4 | ENSG00000196954.12 | 0.77 |
| IFNGR2 | ENSG00000159128.14 | 0.77 |
| CASP1 | ENSG00000137752.22 | 0.77 |
| DPYD | ENSG00000188641.12 | 0.77 |
| TICAM2 | ENSG00000243414.5 | 0.76 |
| PYGL | ENSG00000100504.16 | 0.76 |
| ELK3 | ENSG00000111145.7 | 0.76 |
| IKBIP | ENSG00000166130.14 | 0.76 |
| SP100 | ENSG00000067066.16 | 0.76 |
| ZMYM6NB | ENSG00000243749.1 | 0.76 |
| FNDC3B | ENSG00000075420.12 | 0.76 |
| GLMP | ENSG00000198715.11 | 0.76 |
| CD74 | ENSG00000019582.14 | 0.76 |
| IQGAP1 | ENSG00000140575.12 | 0.76 |
| GAPT | ENSG00000175857.8 | 0.76 |
| PQLC3 | ENSG00000162976.12 | 0.76 |
| IL10RB | ENSG00000243646.8 | 0.75 |
| TNFRSF1A | ENSG00000067182.7 | 0.75 |
| SLC7A7 | ENSG00000155465.18 | 0.75 |
| ARHGDIB | ENSG00000111348.8 | 0.75 |
| GIMAP2 | ENSG00000106560.10 | 0.75 |
| APOBEC3D | ENSG00000243811.7 | 0.75 |
| GLB1 | ENSG00000170266.15 | 0.75 |
| TRIM38 | ENSG00000112343.10 | 0.75 |
| OLFML3 | ENSG00000116774.11 | 0.75 |
| ANXA2 | ENSG00000182718.16 | 0.75 |
| HSD3B7 | ENSG00000099377.13 | 0.75 |
| GALM | ENSG00000143891.16 | 0.75 |
| SUMF1 | ENSG00000144455.13 | 0.75 |
| HEXA | ENSG00000213614.9 | 0.74 |
| MYD88 | ENSG00000172936.12 | 0.74 |
| GSTK1 | ENSG00000197448.13 | 0.74 |
| IL13RA1 | ENSG00000131724.10 | 0.74 |
| MGST2 | ENSG00000085871.8 | 0.74 |
| NPC2 | ENSG00000119655.8 | 0.74 |
| RAC2 | ENSG00000128340.14 | 0.74 |
| CECR1 | ENSG00000093072.15 | 0.74 |
| FCER1G | ENSG00000158869.10 | 0.74 |
| MYL12A | ENSG00000101608.12 | 0.74 |
| HLA-DMA | ENSG00000204257.14 | 0.74 |
| ARHGAP18 | ENSG00000146376.10 | 0.74 |
| VASP | ENSG00000125753.13 | 0.74 |
| SPATS2L | ENSG00000196141.12 | 0.74 |
| CFI | ENSG00000205403.12 | 0.73 |
| CAPZA1 | ENSG00000116489.12 | 0.73 |
| CD300C | ENSG00000167850.3 | 0.73 |
| MFSD1 | ENSG00000118855.18 | 0.73 |
| SH3BGRL3 | ENSG00000142669.13 | 0.73 |
| LCTL | ENSG00000188501.11 | 0.73 |
| HLA-DRA | ENSG00000204287.13 | 0.73 |
| ITGB2 | ENSG00000160255.16 | 0.73 |
| HEXB | ENSG00000049860.13 | 0.73 |
| MSN | ENSG00000147065.16 | 0.73 |
| ANXA2P2 | ENSG00000231991.4 | 0.73 |
| GRN | ENSG00000030582.16 | 0.73 |
| FAM114A1 | ENSG00000197712.11 | 0.72 |
| STEAP3 | ENSG00000115107.19 | 0.72 |
| FCGRT | ENSG00000104870.12 | 0.72 |
| RAB27A | ENSG00000069974.15 | 0.72 |
| ALOX5AP | ENSG00000132965.9 | 0.72 |
| HLA-DMB | ENSG00000242574.8 | 0.72 |
| LRRC25 | ENSG00000175489.9 | 0.72 |
| CAST | ENSG00000153113.23 | 0.72 |
| DOK1 | ENSG00000115325.13 | 0.72 |
| SERPINB1 | ENSG00000021355.12 | 0.72 |
| CYP2S1 | ENSG00000167600.13 | 0.72 |
| CSTA | ENSG00000121552.3 | 0.72 |
| LAPTM5 | ENSG00000162511.7 | 0.72 |
| LHFPL2 | ENSG00000145685.13 | 0.72 |
| CTSC | ENSG00000109861.15 | 0.72 |
| CTSB | ENSG00000164733.20 | 0.72 |
| GPR65 | ENSG00000140030.5 | 0.72 |
| TRAM1 | ENSG00000067167.7 | 0.72 |
| CD63 | ENSG00000135404.11 | 0.72 |
| CTBS | ENSG00000117151.12 | 0.72 |
| LPXN | ENSG00000110031.12 | 0.72 |
| CPQ | ENSG00000104324.15 | 0.72 |
| CARD16 | ENSG00000204397.7 | 0.72 |
| PLBD1 | ENSG00000121316.10 | 0.72 |
| CAPG | ENSG00000042493.15 | 0.71 |
| UNC93B1 | ENSG00000110057.7 | 0.71 |
| CTSZ | ENSG00000101160.13 | 0.71 |
| SH2B3 | ENSG00000111252.10 | 0.71 |
| LYN | ENSG00000254087.7 | 0.71 |
| TRIM34 | ENSG00000258659.6 | 0.71 |
| SLC39A1 | ENSG00000143570.17 | 0.71 |
| PTPN22 | ENSG00000134242.15 | 0.71 |
| EDEM2 | ENSG00000088298.12 | 0.71 |
| CD53 | ENSG00000143119.12 | 0.71 |
| RAP2B | ENSG00000181467.3 | 0.71 |
| TGFB1 | ENSG00000105329.9 | 0.71 |
| NCF1 | ENSG00000158517.13 | 0.71 |
| PDCD1LG2 | ENSG00000197646.7 | 0.71 |
| LAIR1 | ENSG00000167613.15 | 0.71 |
| RGS19 | ENSG00000171700.13 | 0.71 |
| ARL11 | ENSG00000152213.3 | 0.71 |
| SLC10A3 | ENSG00000126903.15 | 0.71 |
| CD151 | ENSG00000177697.17 | 0.71 |
| CHPF2 | ENSG00000033100.14 | 0.71 |
| REXO2 | ENSG00000076043.9 | 0.71 |
| C1RL | ENSG00000139178.10 | 0.71 |
| SEC24D | ENSG00000150961.14 | 0.71 |
| SCIMP | ENSG00000161929.14 | 0.71 |
| ADPGK | ENSG00000159322.17 | 0.71 |
| LGALS1 | ENSG00000100097.11 | 0.71 |
| PDIA4 | ENSG00000155660.10 | 0.71 |
| FUCA2 | ENSG00000001036.13 | 0.71 |
| CMTM6 | ENSG00000091317.7 | 0.71 |
| TLR1 | ENSG00000174125.7 | 0.71 |
| SASH3 | ENSG00000122122.9 | 0.71 |
| ABHD15 | ENSG00000168792.4 | 0.7 |
| HLA-DRB1 | ENSG00000196126.10 | 0.7 |
| SERPING1 | ENSG00000149131.15 | 0.7 |
| LIMS1 | ENSG00000169756.16 | 0.7 |
| MSR1 | ENSG00000038945.14 | 0.7 |
| LCP2 | ENSG00000043462.11 | 0.7 |
| CCDC109B | ENSG00000005059.15 | 0.7 |
| SP140L | ENSG00000185404.16 | 0.7 |
| CTSA | ENSG00000064601.16 | 0.7 |
| SH3GLB1 | ENSG00000097033.14 | 0.7 |
| OSCAR | ENSG00000170909.13 | 0.7 |
| GALNT2 | ENSG00000143641.9 | 0.7 |
| FGL2 | ENSG00000127951.5 | 0.7 |
| CXCL16 | ENSG00000161921.14 | 0.7 |
| SERPINA1 | ENSG00000197249.12 | 0.7 |
| LOXL3 | ENSG00000115318.11 | 0.7 |
| RNASE6 | ENSG00000169413.2 | 0.7 |
| PPP1R18 | ENSG00000146112.11 | 0.7 |
| TMEM109 | ENSG00000110108.9 | 0.7 |
| SNX20 | ENSG00000167208.14 | 0.7 |
| ICAM3 | ENSG00000076662.9 | 0.7 |
| NAT1 | ENSG00000171428.13 | 0.7 |
| TMED9 | ENSG00000184840.11 | 0.7 |
| ZNRF2 | ENSG00000180233.10 | 0.7 |
| PIGT | ENSG00000124155.16 | 0.7 |
| MILR1 | ENSG00000271605.5 | 0.7 |
| TYROBP | ENSG00000011600.11 | 0.7 |
| PFN1 | ENSG00000108518.7 | 0.7 |
| NMI | ENSG00000123609.10 | 0.7 |
| FCGR3A | ENSG00000203747.9 | 0.7 |
| PVRL2 | ENSG00000130202.9 | 0.7 |
| HLA-DPB1 | ENSG00000223865.10 | 0.7 |
| LY75 | ENSG00000054219.10 | 0.69 |
| PYCARD | ENSG00000103490.13 | 0.69 |
| MRC2 | ENSG00000011028.13 | 0.69 |
| FAH | ENSG00000103876.11 | 0.69 |
| PTRF | ENSG00000177469.12 | 0.69 |
| SPI1 | ENSG00000066336.11 | 0.69 |
| MOB1A | ENSG00000114978.17 | 0.69 |
| GPSM3 | ENSG00000213654.9 | 0.69 |
| B2M | ENSG00000166710.17 | 0.69 |
| EFEMP2 | ENSG00000172638.12 | 0.69 |
| SSR3 | ENSG00000114850.6 | 0.69 |
| SLC2A9 | ENSG00000109667.11 | 0.69 |
| ARHGAP15 | ENSG00000075884.12 | 0.69 |
| PDIA3 | ENSG00000167004.12 | 0.69 |
| DENND2D | ENSG00000162777.16 | 0.69 |
| SRPX2 | ENSG00000102359.5 | 0.69 |
| ACTB | ENSG00000075624.13 | 0.69 |
| ARPC2 | ENSG00000163466.15 | 0.69 |
| PDIA6 | ENSG00000143870.12 | 0.69 |
| TAGLN2 | ENSG00000158710.14 | 0.69 |
| LPAR6 | ENSG00000139679.15 | 0.69 |
| TRIP6 | ENSG00000087077.11 | 0.69 |
| CALHM2 | ENSG00000138172.10 | 0.69 |
| FCGR2A | ENSG00000143226.13 | 0.69 |
| DDB2 | ENSG00000134574.11 | 0.69 |
| SPPL2A | ENSG00000138600.9 | 0.69 |
| CYBA | ENSG00000051523.10 | 0.69 |
| LY96 | ENSG00000154589.6 | 0.69 |
| TSPO | ENSG00000100300.17 | 0.69 |
| GLIPR1 | ENSG00000139278.9 | 0.69 |
| MBD2 | ENSG00000134046.11 | 0.69 |
| LGALS3BP | ENSG00000108679.12 | 0.69 |
| PSMC2 | ENSG00000161057.10 | 0.69 |
| ELF4 | ENSG00000102034.16 | 0.69 |
| SEPN1 | ENSG00000162430.16 | 0.69 |
| VAMP5 | ENSG00000168899.4 | 0.69 |
| CASP6 | ENSG00000138794.9 | 0.69 |
| CYB561A3 | ENSG00000162144.9 | 0.69 |
| ARHGAP9 | ENSG00000123329.17 | 0.69 |
| ADAM9 | ENSG00000168615.11 | 0.69 |
| CLDN23 | ENSG00000253958.1 | 0.69 |
| TNFSF8 | ENSG00000106952.7 | 0.69 |
| NCF2 | ENSG00000116701.14 | 0.69 |
| TRIM21 | ENSG00000132109.9 | 0.68 |
| TGFBR2 | ENSG00000163513.17 | 0.68 |
| TFEC | ENSG00000105967.15 | 0.68 |
| RPS6KA1 | ENSG00000117676.13 | 0.68 |
| RP11-121A8.1 | ENSG00000272908.1 | 0.68 |
| PCED1B | ENSG00000179715.12 | 0.68 |
| RIPK1 | ENSG00000137275.13 | 0.68 |
| GUSB | ENSG00000169919.16 | 0.68 |
| KDELC2 | ENSG00000178202.12 | 0.68 |
| CD86 | ENSG00000114013.15 | 0.68 |
| EMP3 | ENSG00000142227.10 | 0.68 |
| CPVL | ENSG00000106066.13 | 0.68 |
| KDELR2 | ENSG00000136240.9 | 0.68 |
| CTSS | ENSG00000163131.10 | 0.68 |
| CACNA2D4 | ENSG00000151062.14 | 0.68 |
| B3GNT5 | ENSG00000176597.11 | 0.68 |
| RP11-342M1.3 | ENSG00000228192.7 | 0.68 |
| CD4 | ENSG00000010610.9 | 0.68 |
| AC109826.1 | ENSG00000226791.6 | 0.68 |
| HAVCR2 | ENSG00000135077.8 | 0.68 |
| PARP9 | ENSG00000138496.16 | 0.68 |
| LAP3 | ENSG00000002549.12 | 0.68 |
| CYBB | ENSG00000165168.7 | 0.68 |
| ARHGAP25 | ENSG00000163219.11 | 0.68 |
| SLC43A3 | ENSG00000134802.17 | 0.68 |
| RBMS1 | ENSG00000153250.17 | 0.68 |
| GPR141 | ENSG00000187037.8 | 0.68 |
| CRTAP | ENSG00000170275.14 | 0.68 |
| DOK3 | ENSG00000146094.13 | 0.68 |
| CD164 | ENSG00000135535.14 | 0.68 |
| FERMT3 | ENSG00000149781.12 | 0.68 |
| IKBKE | ENSG00000263528.7 | 0.68 |
| ALOX5 | ENSG00000012779.10 | 0.68 |
| LAT2 | ENSG00000086730.16 | 0.68 |
| RUNX1 | ENSG00000159216.18 | 0.68 |
| ALPK1 | ENSG00000073331.17 | 0.68 |
| TMEM106A | ENSG00000184988.8 | 0.68 |
| NCF1C | ENSG00000165178.9 | 0.67 |
| C16orf54 | ENSG00000185905.3 | 0.67 |
| C1R | ENSG00000159403.15 | 0.67 |
| CD68 | ENSG00000129226.13 | 0.67 |
| PPIC | ENSG00000168938.5 | 0.67 |
| HLA-DPA1 | ENSG00000231389.7 | 0.67 |
| P2RX4 | ENSG00000135124.14 | 0.67 |
| GPX8 | ENSG00000164294.13 | 0.67 |
| MYOF | ENSG00000138119.16 | 0.67 |
| CD58 | ENSG00000116815.15 | 0.67 |
| ZDHHC12 | ENSG00000160446.18 | 0.67 |
| BACE2 | ENSG00000182240.15 | 0.67 |
| SYDE1 | ENSG00000105137.12 | 0.67 |
| TMSB4X | ENSG00000205542.10 | 0.67 |
| ENTPD1 | ENSG00000138185.16 | 0.67 |
| SOAT1 | ENSG00000057252.12 | 0.67 |
| TRIM6 | ENSG00000121236.19 | 0.67 |
| ITGB1 | ENSG00000150093.18 | 0.67 |
| ITGB1P1 | ENSG00000269378.1 | 0.67 |
| ATP6V0E1 | ENSG00000113732.8 | 0.67 |
| DAP | ENSG00000112977.15 | 0.67 |
| NCF1B | ENSG00000182487.12 | 0.67 |
| AGTRAP | ENSG00000177674.15 | 0.67 |
| SLC15A3 | ENSG00000110446.9 | 0.67 |
| SHISA5 | ENSG00000164054.15 | 0.67 |
| MGAT1 | ENSG00000131446.15 | 0.67 |
| CD276 | ENSG00000103855.17 | 0.67 |
| ADAP2 | ENSG00000184060.10 | 0.67 |
| ZNF217 | ENSG00000171940.13 | 0.67 |
| PTPN12 | ENSG00000127947.15 | 0.67 |
| COLGALT1 | ENSG00000130309.10 | 0.67 |
| TMEM154 | ENSG00000170006.11 | 0.67 |
| PCED1B-AS1 | ENSG00000247774.6 | 0.67 |
| IFI30 | ENSG00000216490.3 | 0.67 |
| C5orf15 | ENSG00000113583.7 | 0.67 |
| CD300LF | ENSG00000186074.18 | 0.67 |
| ANXA4 | ENSG00000196975.14 | 0.67 |
| SWAP70 | ENSG00000133789.14 | 0.67 |
| LILRB2 | ENSG00000131042.13 | 0.67 |
| COPZ2 | ENSG00000005243.9 | 0.67 |
| CNPY4 | ENSG00000166997.7 | 0.67 |
| LMAN2 | ENSG00000169223.14 | 0.67 |
| TUBA1C | ENSG00000167553.14 | 0.67 |
| VAMP8 | ENSG00000118640.10 | 0.67 |
| PLSCR1 | ENSG00000188313.12 | 0.67 |
| AC011899.9 | ENSG00000233038.5 | 0.67 |
| RHOH | ENSG00000168421.12 | 0.67 |
| VIM | ENSG00000026025.13 | 0.67 |
| PTPN6 | ENSG00000111679.16 | 0.67 |
| FHL3 | ENSG00000183386.9 | 0.67 |
| RER1 | ENSG00000157916.18 | 0.67 |
| CROT | ENSG00000005469.11 | 0.67 |
| FBXO22 | ENSG00000167196.13 | 0.67 |
| CARD8-AS1 | ENSG00000268001.1 | 0.66 |
| ORMDL2 | ENSG00000123353.9 | 0.66 |
| BRI3 | ENSG00000164713.9 | 0.66 |
| SLC2A10 | ENSG00000197496.5 | 0.66 |
| RBM47 | ENSG00000163694.14 | 0.66 |
| TWF2 | ENSG00000247596.8 | 0.66 |
| CALD1 | ENSG00000122786.19 | 0.66 |
| MMAA | ENSG00000151611.13 | 0.66 |
| LSP1 | ENSG00000130592.13 | 0.66 |
| CMTM3 | ENSG00000140931.19 | 0.66 |
| UBE2F | ENSG00000184182.18 | 0.66 |
| OSMR | ENSG00000145623.12 | 0.66 |
